# Gallic acid: a dietary metabolite’s therapeutic potential in the management of atherosclerotic cardiovascular disease

**DOI:** 10.3389/fphar.2024.1515172

**Published:** 2025-01-07

**Authors:** Xiao-Lan Zhao, Zhang-Jing Cao, Ke-Di Li, Fei Tang, Li-Yue Xu, Jing-Nan Zhang, Dong Liu, Cheng Peng, Hui Ao

**Affiliations:** ^1^ State Key Laboratory of Southwestern Chinese Medicine Resources, Chengdu University of Traditional Chinese Medicine, Chengdu, China; ^2^ Innovative Institute of Chinese Medicine and Pharmacy, Chengdu University of Traditional Chinese Medicine, Chengdu, China

**Keywords:** gallic acid, diabetes, hypertension, hyperlipidemia, atherosclerosis, ASCVD, cardio-vascular diseases

## Abstract

Atherosclerotic cardiovascular disease (ASCVD) causes significant morbidity and mortality globally. Most of the chemicals specifically target certain pathways and minimally impact other diseases associated with ASCVD. Moreover, interactions of these drugs can cause toxic reactions. Consequently, the exploration of multi-targeted and safe medications for treating and preventing ASCVD has become an increasingly popular trend. Gallic acid (GA), a natural secondary metabolite found in various fruits, plants, and nuts, has demonstrated potentials in preventing and treating ASCVD, in addition to its known antioxidant and anti-inflammatory effects. It alleviates the entire process of atherosclerosis (AS) by reducing oxidative stress, improving endothelial dysfunction, and inhibiting platelet activation and aggregation. Additionally, GA can treat ASCVD-related diseases, such as coronary heart disease (CHD) and cerebral ischemia. However, the pharmacological actions of GA in the prevention and treatment of ASCVD have not been comprehensively reviewed, which limits its clinical development. This review primarily summarizes the *in vitro* and *in vivo* pharmacological actions of GA on the related risk factors of ASCVD, AS, and ASCVD. Additionally, it provides a comprehensive overview of the toxicity, extraction, synthesis, pharmacokinetics, and pharmaceutics of GA,aimed to enhance understanding of its clinical applications and further research and development.

## 1 Introduction

According to the latest statistics, the absolute number of CVD incident cases and deaths remains an increasing worldwide (Li et al., 2023). It is projected that by 2050, In the United States, 61% of adults will have some form of cardiovascular disease, primarily due to increases in hypertension, obesity, and diabetes (Joynt Maddox et al., 2024). And ischaemic heart disease will remain the leading cause of cardiovascular deaths (20 million deaths) (Chong et al., 2024). Atherosclerotic cardiovascular disease (ASCVD) is a major type of cardiovascular disease (CVD), with risk factors that include hypertension, high cholesterol, smoking, diabetes, and obesity. Preventive measures should involve lifestyle modifications, such as maintaining a healthy diet, engaging in regular physical activity, and quitting smoking, as well as pharmacological interventions to control blood pressure, blood glucose, and lipid levels (Al Rifai et al., 2022; Pasquel et al., 2018; Qiao et al., 2022; Zhou et al., 2023).

However, traditional risk assessment tools tend to underestimate the risk for high-risk populations, while the application of emerging technologies, lifestyle interventions, and personalized treatments is hindered by issues such as cost, resources, and adherence to treatment. There is an urgent need for innovative, safer, and more accessible strategies to improve prevention and management. However, the treatment of acute cardiovascular diseases often involves multiple medications, which can lead to side effects and toxicity (Barkas et al., 2024). In this context, natural multi-target drugs such as quercetin, berberine, and curcumin exhibit significant commercial potential (Chen et al., 2020; Deng et al., 2020; Lv et al., 2024). These natural products, distinguished from synthetic chemicals, hold promise as alternative options for the prevention and treatment of ASCVD, owing to their low toxicity and multifunctional effects. Therefore, exploring natural compounds for the prevention and treatment of ASCVD is of critical importance, as it holds the potential to offer substantial benefits in managing this condition.

GA (3,4,5-trihydroxybenzoic acid), first identified by Scheele in 1786 and derived from oak apple extract, is a natural secondary metabolite predominantly extracted from various fruits, plants, and nuts (Wianowska and Olszowy-Tomczyk, 2023). This compound is extensively utilized as a food preservative, in brewing, cosmetics, and food packaging (Limpisophon and Schleining, 2017).

GA exhibited multi-target and multi-level pathophysiological effects, demonstrating significant therapeutic potential in several key pathological processes of ASCVD. In addition to its antioxidant and anti-inflammatory properties, GA regulated lipid metabolism to lower cholesterol levels, improved insulin sensitivity, reduced blood glucose levels, and decreased systolic blood pressure, thereby mitigating the risk of acute cardiovascular events. Furthermore, GA enhanced endothelial function, inhibited platelet aggregation and thrombosis, reduced abnormal vascular smooth muscle cell proliferation, and stabilized atherosclerotic plaques, comprehensively addressing the pathological processes of ASCVD. These characteristics underscored GA’s immense potential in the prevention and treatment of ASCVD, positioning it as a promising natural candidate for further research and development (Choubey et al., 2018; Lekakis et al., 2005).However, these findings have not been systematically summarized, which will limit the clinical application and further development of GA.

Consequently, this review systematically assesses the research on the therapeutic potential of GA across various stages of ASCVD progression, pointing out that GA is a promising drug candidate applied in the prevention and treatment of ASCVD. We anticipate that this comprehensive review will further facilitate development and application of GA as a therapeutic agent against ASCVD.

## 2 Development and research of GA

GA possesses a wide range of pharmacological effects and has demonstrated potential therapeutic value in various aspects of ASCVD. In this section, the availability, pharmacokinetics, and pharmacodynamics of GA were summarized.

### 2.1 Availability of GA

#### 2.1.1 Extraction of GA

GA, a phenolic compound, is found in various plants, including black tea, green tea, quince, pomegranate, oranges, grapes, and berries. Given the wide range of sources containing GA, various extraction techniques, including hot water extraction, supercritical CO2 extraction, solid-phase extraction, and ultrasonic-assisted extraction, have been developed (Du et al., 2009; Murga et al., 2000; Palma et al., 1999; Pawar and Surana, 2010).

Researchers have found that hot water extraction is the primary method for extracting antioxidants. Pawar et al. optimized the extraction parameters forextracting GA from *Caesalpinia decapetala*. The ideal conditions were an extraction temperature of 65–70°C, an extraction period of 48 h, and a solvent mixture of ethanol to water (70:30). Under these optimal conditions, the maximum yield of GA was 17.85% (Pawar and Surana, 2010). However, the extraction of GA from *Emblica officinalis,* by the preparation of molecularly imprinted microspheres and nanoparticles through precipitation polymerization and subsequent elution with hot water, significantly increased the extraction yield to 28% (Pardeshi et al., 2014). Additionally, GA was extracted from pomegranate rind using solid-phase extraction with surface-imprinted polymers on magnetic carbon nanotubes with a yield of 3.16 mg/g (Hao et al., 2015). The optimum extraction yield of GA (8.57 mg/g) was achieved by preparing hydrophilic molecularly imprinted chitosan and employing solid-phase microextraction methods, using the response surface methodology strategy (Li and Row, 2019). Khodaie et al. successfully extracted GA (50.54%) from the seeds of the sumac species *Rhus coraria* using supercritical CO_2_ with ethanol as a co-solvent (Khodaie and Ghoreishi, 2021). In addition, when GA was extracted from the leaves of *Ficus auriculata*, alkaline water and ultrasonic-assisted extraction were used. This method was environmentally friendly and safe. The results showed that the extraction yield with weak alkaline water was 284.2 mg/L, second only to 50% methanol (Baite et al., 2021).

#### 2.1.2 Synthesis of GA

It have been found that GA was synthesized through the shikimic acid pathway (Fernandes and Salgado, 2016; Wu et al., 2022; Zhang et al., 2009). Researches showed that shikimate dehydrogenase (SDH) was an essential enzyme in the shikimate pathway for GA synthesis. The aroE mutant strain of *Escherichia coli* could produce GA through functional complementation with plant-derived SDH. In transgenic tobacco (*Nicotiana tabacum*) expressing SDH from walnut (*Juglans regia*), accumulation of GA was increased by 500% (Muir et al., 2011). Additionally, with the high-yield, high-titer synthesis of 3-dehydroshikimic acid from glucose using recombinant *E. coli*, GA was efficiently synthesized from 3-dehydroshikimic acid by choosing the appropriate solvent (such as acetic acid) and optimizing the catalytic system (such as using Cu (OAc)_2_ and ZnO). And the yield of GA was significantly increased to 67% (Kambourakis and Frost, 2000).

Additionally, in nature or within plants, GA was synthesized through the degradation of tannic acid via tannase, a glycoprotein esterase. This enzyme was produced by fermentation with various microorganisms, particularly fungi from the *Aspergillus* and *Penicillium* genera (Aguilar-Zárate et al., 2015; Dhiman et al., 2018). For example, the novel *Penicillium roqueforti* strain could produce both tannase and GA simultaneously (Andrade et al., 2018). Moreover, the marine *Aspergillus awamori* strain BTMFW032, under deep fermentation conditions, was able to simultaneously produce GA and tannase, resulting in a 15-fold increase in the yields of both tannase and GA (Beena et al., 2011). Furthermore, other microorganisms such as *Rhodotorula pilimanae* A45.2 and *Bacillus* spheroides have also been noted for their abilities to co-produce GA and tannase, with the latter achieving high efficiency in converting tannic acid to GA (90.80% crystallization), making it the most potent bacterial tannase producer for GA synthesis (Kanpiengjai et al., 2020; Raghuwanshi et al., 2011). Recent research discovered that a high-activity PobA variant, Y385F/T294A-PobA, was developed from the hydroxyphenylacetic acid hydroxylase of *Pseudomonas aeruginosa*. This variant exhibited a molar conversion rate of up to 93%, providing a promising pathway for the biomanufacturing of GA and its derivatives (Chen et al., 2017). In summary, GA is primarily synthesized through the shikimic acid pathway, and in some cases, it is also produced as a by-product of tannic acid decomposition.

### 2.2 Pharmacokinetics of GA

It is well known that GA has a wide range of sources, and different forms of GA exhibit significant differences in pharmacokinetic parameters. Studies found that compared to pure GA, the half-life (T_1/2_) (93.72–128.52 min) and time to reach maximum concentration (T_max_) (40–100 min) of GA in P. capitatum extracts were prolonged (Ma et al., 2015). After oral administration of Japanese toad venom extract SD in rats, the pharmacokinetics parameters for GA were determined using a single-compartment model. It was found that T_max_, maximum plasma concentration (C_max_) T_1/2_, AUC_0-∞,_ in SD rats were 1.40 ± 1.13 h, 35.5 ± 10.2 ng/mL, 3.21 ± 4.56 h, 199.7 ± 43.9 ng/mL*h (Yu X.-A. et al., 2018). After oral administration of *Hedyotis diffusa* Willd extract to SD rats, the T_1/2_, C_max_ and AUC_0–∞_ of GA were 1.54 ± 0.43h, 919.48 ± 35.03 ng/mL, 8213.20 ± 733.40 ng•h/mL (Chen et al., 2018a). Additionally, after administration of a single oral dose of tea (containing 0.3 mmol of GA) to 10 volunteers, T_1/2_ was 1.06 ± 0.06 h, and C_max_ was 2.09 ± 0.22 μmol/L (Shahrzad et al., 2001). Pharmacokinetic differences between GA and GA-loaded carboxymethylchitosan nanoparticles (GANPs) were examined in SD rats after gastric administration. Nanoparticles significantly improved area under the curve (AUC) (13.8 mg/min/mL and 5.2 mg/min/mL) and T_1/2_ (2.0 h and 2.7 h) of GA (Y. Zhao et al., 2020a). Additionally, dosage also affected GA absorption. Repeated daily exposure to grape seed polyphenol extract (GSPE) significantly increased the bioavailability of GA. When the GSPE dose reached 100 mg/kg BW, AUC_0–8h_ of GA significantly was increased from 512.7 to 673 ng/mL•h (Ferruzzi et al., 2009). The AUC_0–8h_ (area under the concentration-time curve from 0 to 8 h) was used to evaluate the pharmacokinetics of the compound (Alpízar et al., 2024). Yu et al. found that pathological conditions can alter the pharmacokinetics of GA. In the study, compared to normal rats, the absorption rate of GA in rats with myocardial infarction was slower. When the dose of GA was 100 mg/kg, the AUC decreased by approximately 23%. The C_max_ was reduced by 2.5 times, and the half-life was significantly prolonged (Yu Z. et al., 2018). In summary, absorption of GA was altered by different administration route, dosage, and health status.

Also, GA was extensively distributed across various tissues, predominantly in the kidneys, followed by the heart, liver, spleen, and lungs (Chen et al., 2018b). Notably, it was primarily localized in the kidneys and liver (Liu et al., 2019). FW et al. reported that GA showed a targeted distribution in renal tissues, achieving a concentration of 1218.62 ng/g 1 hour post-administration of 60 mg/kg of Cephalonia extract (equivalent to 12 mg/kg GA)(Ma et al., 2016; Wei et al., 2020).In addition, researches found that the metabolites of GA underwent typical methylation reactions in the liver, which increased the polarity of the molecules, thereby facilitating their excretion in the kidneys. High-performance liquid chromatography (HPLC) analysis revealed that GA was metabolized into several structurally similar compounds, including pyrogallol, 2-*O*-methyl-phenylenetriol, and 4-O-methylgallic acid (Yasuda et al., 2000). Additional researches by FW et al. on the urinary excretion of *Pseudomonas cephalosporium* extracts indicated that GA underwent significant metabolism, primarily to 4-methyl GA (4-Ω) and 4-methyl protocatechuic acid (4-OMePCA) (Hsu C.-L. et al., 2007). Other predominant mammalian metabolites included 3-O-methyl GA, 4-O-methyl GA, and 3,4-O-dimethyl GA (Hodgson et al., 2000). High-performance liquid chromatography analyses demonstrated that GA was predominantly metabolized into 4-O-methyl GA in peripheral blood and urine (Shahrzad and Bitsch, 1998). Overall, GA was absorbed through the gastrointestinal tract, primarily distributed in the kidneys, then metabolized in the liver, and excreted by the kidneys.

### 2.3 Pharmaceutics of GA

The development of various formulations has enhanced the controlled release, bioavailability, and therapeutic efficacy of GA under different disease conditions. The utilization of contemporary technologies and encapsulation methodologies has facilitated the development of an array of formulations of GA, encompassing nanoparticles, hydrogels, gels, inclusion complexes, microcapsules, nanoemulsions and liposomes.

Killedar et al. found that nanoparticles effectively controlled drug release, thereby enhancing the bioavailability of GA. Formulating GA into chitosan nanoparticles effectively controls the release of the drug, achieving an accumulative *in vitro* release rate of 77.16% for GA (Patil and Killedar, 2021a). The modification of chitosan nanoparticles with hyaluronic acid resulted in the creation of HA@CS-GA NPs, which proved to be an effective treatment for recalcitrant skin diseases such as psoriasis (Sheikh et al., 2023). Further studies developed a lipid-polymer hybrid nanoparticle system (LPHNs) containing GA, which penetrated deeper layers of the skin more effectively, demonstrating a higher drug release rate of 79% ± 0.001% (Hazari et al., 2023). Encapsulating GA into nanoparticles using gum arabic (GANPs) enhanced its bioavailability (Hassani et al., 2020). Additionally, the combination of GA with magnetite nanoparticles constituted an innovative nanotechnology-based drug formulation that, with the assistance of an external magnetic field, crossed the blood-brain barrier (Azarmi et al., 2023).

In addition, gels and hydrogels effectively control drug release and are mostly used for dermal administration. Research has found that the gel formulations of GA were commonly used in cosmetics, reducing lipid peroxidation by 33.97%, which confirmed their antioxidant effects in the skin’s stratum corneum (Monteiro E Silva et al., 2017). Additionally, research has developed and characterized a poloxamer gel containing the antioxidant molecule GA, capable of precisely controlling drug release and enhancing the local concentration of the drug at the target site, typically used for topical administration in melanoma skin treatments (Sguizzato et al., 2020). Additionally, Hydrogels based on chitosan (CS) and 2-acrylamido-2-methylpropane sulfonic acid (AMPS) were prepared using free radical polymerization techniques for the controlled release of GA (Yu et al., 2022). Hydrogel films based on sodium alginate and a polyvinyl alcohol-acrylic acid copolymer loaded with GA were also used for skin wound healing (Naeem et al., 2022). Furthermore, the water absorption rate of hydrogels loaded with GA-based carbon nanoparticles (GACNPs) was significantly higher than that of blank hydrogels, demonstrating a desirable characteristic for wound dressing applications (Dechsri et al., 2024).

Besides this, other GA-related formulations can also control drug release and enhance drug delivery efficiency and bioavailability. Inclusion complexes formed by ferulic acid (FA) and GA with 2-hydroxypropyl-β-cyclodextrin (HPβCD) through spray drying techniques have enhanced drug release and bioavailability (Patil and Killedar, 2021b). Erik and colleagues found that encapsulating GA in a polymer matrix composed of sodium alginate and pectin provided an alternative method for protecting and controlling the release of GA (Nájera-Martínez et al., 2023). Furthermore, the developed self-nanoemulsifying drug delivery system (SNEDDS) loaded with GA offered an effective method for enhancing the transdermal delivery efficiency of poorly soluble drugs (Khan et al., 2024). Moreover, GA was encapsulated in stealth liposomes, which were functionalized with transferrin (Tf) to deliver the drug directly to the brain for sustained release (Andrade et al., 2022). A novel co-loaded nanoliposome system containing GA and quercetin was developed, enhancing the stability and bioavailability of the drugs in the body (Al-Samydai et al., 2023).

In summary, the use of various formulation technologies has enabled the precise and effective application of GA across different areas, adding significant value to its clinical use.

## 3 Therapeutic potential of GA for the pathogenetic basis of ASCVD

Hyperlipidemia, arterial stiffening, and AS collectively constitute the core pathological basis of ASCVD (Dutta et al., 2024; Hoshino et al., 2024; Szołtysek-Bołdys et al., 2024). This process begins with early lipid metabolism abnormalities, gradually leading to vascular dysfunction and structural changes, ultimately resulting in the onset and progression of ASCVD. GA, through its inhibitory effects on hepatic cholesterol synthesis, multifaceted vascular protection, and its role in suppressing atherosclerosis, offered a novel strategy for the comprehensive intervention of ASCVD.

### 3.1 Hypolipidemic effect of GA

**FIGURE 1:**
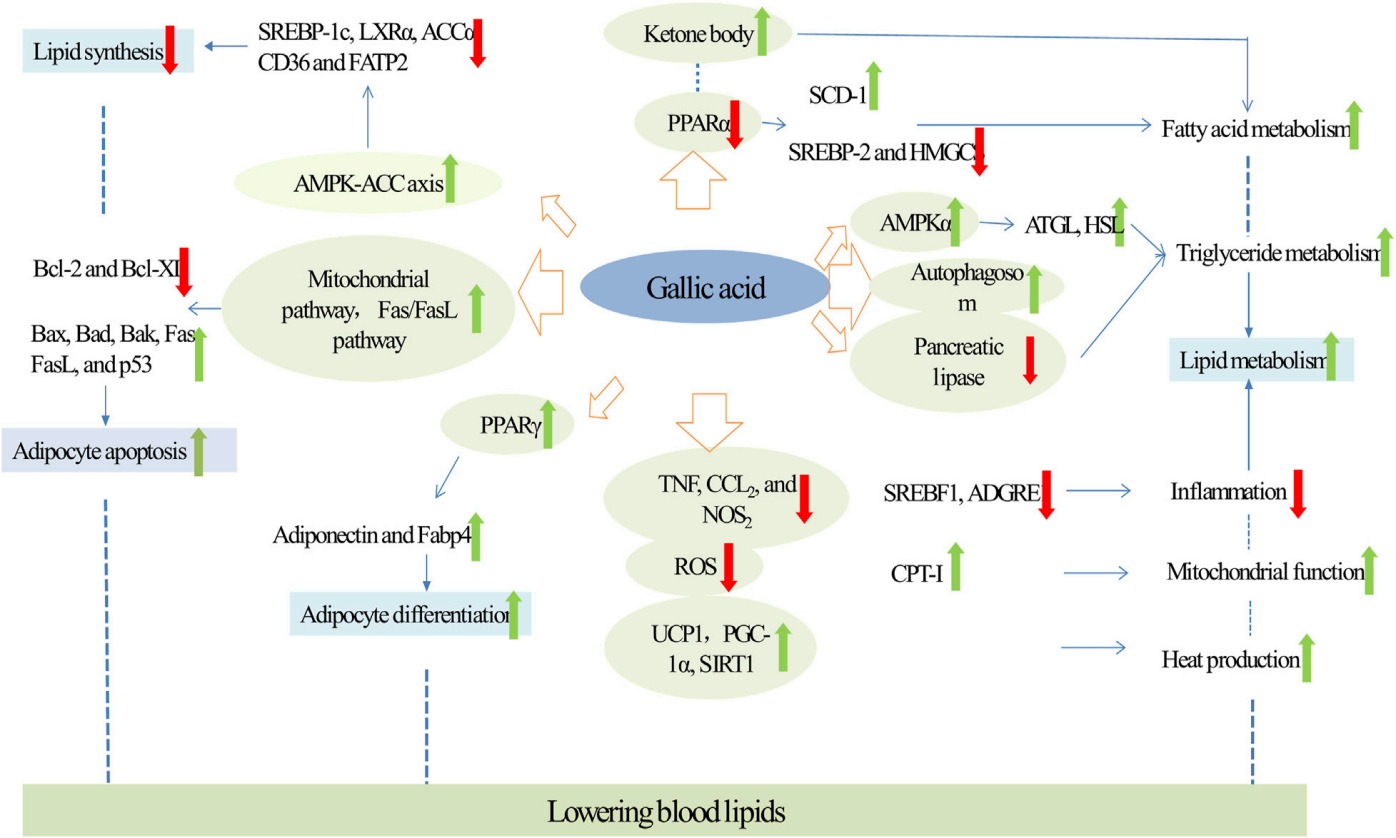
Molecular targets and mechanism of action of GA in hyperlipidemia. Green arrows and red arrows indicate promotion and inhibition, respectively. Description: This figure illustrates how GA regulates lipid metabolism in patients with hyperlipidemia. GA inhibits lipid synthesis by activating the AMPK pathway, downregulating SREBP-1c, SREBP-2, and ACCα. Simultaneously, it promotes fatty acid oxidation by decreasing PPARα expression and increasing ketone body levels. GA also reduces triglyceride accumulation by activating AMPKα, promoting autophagy, and inhibiting HSL and pancreatic lipase. Furthermore, GA suppresses inflammation by reducing TNF, CCL-2, and NOS levels, while enhancing mitochondrial function through UCP1, PGC-1α, and SIRT1. Additionally, GA induces adipocyte apoptosis by inhibiting the expression of Bcl-2 and Bcl-XL and promotes adipocyte differentiation, leading to increased production of adipokines such as adiponectin and Fabp4, thereby indirectly influencing lipid levels. These combined effects underscore GA’s potential for effective control of hyperlipidemia. Green arrows indicate promoting effects, while red arrows represent inhibitory actions.

Recent studies have revealed that hyperlipidemia, characterized by elevated levels of low-density lipoprotein cholesterol, very-low-density lipoprotein cholesterol, and apolipoprotein B (ApoB), directly promotes the onset and progression of atherosclerosis, making it a key pathogenic factor in ASCVD (Correction, 2023). Studies have demonstrated that GA, a natural compound, effectively improves lipid profiles by inhibiting lipid synthesis, promoting lipid metabolism and adipocyte differentiation, and inducing adipocyte apoptosis, thereby exerting a substantial lipid-lowering effect (Figure 1).

#### 3.1.1 Inhibiting lipid synthesis

It has been established that GA can ameliorate lipid accumulation by inhibiting lipogenesis, a critical strategy for lowering lipid levels. GA reduced the activity of transcription factors involved in lipid synthesis. Particularly noteworthy is its targeting of the acetyl-CoA carboxylase (ACC) family, which acts as a rate-limiting enzyme in fatty acid metabolism, thereby reducing overall lipid synthesis (Chen et al., 2019; Yeudall et al., 2022). GA strongly activated the AMPK-ACC axis in a dose-dependent manner, thereby inhibiting lipid synthesis. In mice fed high-fat diet (HFD), GA reduced the mRNA expressions of the lipidogenesis marker ACC, FAS (Fatty Acid Synthase)/B-actin, ACC/B-actin and Acetyl-CoA Carboxylase Beta (Sousa et al., 2020). Futhermore, in HepG2 cells, GA treatment activated AMPK signaling and reduced mRNA expression of key transcription factors in fatty acid synthesis, including SREBP-1c, LXRα, and ACCα, as well as fatty acid transporter proteins such as CD36 and FATP2, which mitigated palmitic acid-induced lipid accumulation (Tanaka et al., 2020a). In summary, GA primarily inhibited lipogenesis and improved lipid profiles through the AMPK pathway.

#### 3.1.2 Promoting lipid metabolism

Researches have demonstrated that GA mitigated lipid accumulation by enhancing lipid metabolism which involved s several key pathways.

On one hand, GA directly promoted lipid metabolism. Firstly, GA treatment promoted fatty acid metabolism. Specifically, GA increased beta-oxidation of fatty acids and elevated levels of ketone body metabolites which are produced during fatty acid catabolism. This process enhanced lipolysis, thereby reducing hepatic cholesterol accumulation. In mice with HFD-induced non-alcoholic fatty liver disease (NAFLD), GA treatment raised ketone body levels in both serum and urine, enhanced beta-oxidation in the liver, and reduced excessive fat accumulation in intracellular vacuoles. Additionally, GA treatment decreased liver triglyceride (TG), cholesterol, and fatty acid levels (Chao et al., 2014a). Peroxisome proliferator-activatedve receptor alpha (PPARα) regulates genes involved in lipid metabolism, influencing lipid homeostasis (Bougarne et al., 2018). In HFD mice, GA could also significantly reduce the expression of PPARα. It downregulated the expression of the lipogenic enzyme monounsaturated fatty acid synthase (SCD-1) and upregulated the expressions of key genes for triglyceride synthesis, such as sterol regulatory element-binding protein 2 (SREBP-2) and the cholesterol synthesis gene β-hydroxy-β-methylglutaryl-CoA synthase (HMGCS), which led to the inhibition of cholesterol synthesis and the reduction of lipid accumulation (Chao et al., 2020; Lee et al., 2021).

Secondly, GA treatment enhanced triglyceride metabolism. Pancreatic lipase was essential for breaking down triglycerides into glycerol and fatty acids, which prevented the absorption and digestion of triglycerides (Modanwal et al., 2024). In diet-induced obese mice, GA inhibited triglyceride uptake and digestion by decreasing pancreatic lipase activity (Oi et al., 2012). Adipose triglyceride lipase (ATGL) and hormone-sensitive lipase (HSL) are key enzymes in lipolysis (Grabner et al., 2021). Studies have shown that GA increased ATGL expression in the perirenal adipose tissue of HFD rats, leading to decreased hypertriglyceridemia and fat accumulation (Huang et al., 2018). In bovine subcutaneous adipocytes, GA reduced TG levels b increasing the expression of lipolysis-related genes such as ATGL and HSL, and activating the metabolic regulator AMPKα (Jin et al., 2022). Moreover, GA promoted triglyceride metabolism by inducing autophagy. According to Singh et al., autophagy is a critical pathway for regulating cellular lipid levels by digesting lipid droplets in autophagic lysosomes, thus reducing TG storage (Grabner et al., 2021). In HepG2 cells, an increase in autophagosome formation, marked by the conversion of LC3-I to LC3-II, led to a reduction in oleic acid (OA)-induced TG accumulation (Doan et al., 2015).

On the other hand, GA treatment can also indirectly enhance lipid metabolism. Firstly, GA treatment can indirectly enhance lipid metabolism by inhibiting inflammation. Increasing evidences suggested that inflammation in adipose tissue triggered lipolysis, leading to the excessive release of free fatty acids and subsequent hepatic lipid accumulation (Badmus et al., 2022; van Dierendonck et al., 2022). By reducing inflammatory responses, GA potentially lowered the risk of abnormal lipid metabolism and related diseases. In dust-exposed HFD-induced rats, GA pretreatment notably reduced the expression of NF-κB, interleukin-6 (IL-6), and tumor necrosis factor-alpha (TNF-α), and the serum levels of triglycerides, cholesterol, and low-density lipoprotein (LDL) (Fanaei et al., 2021). In the white adipose tissue of diet-induced obese mice, GA diminished the expressions of *Il6*, *Nos*_*2*,_ *Ptgs*_*2*,_ *Adgre*_*1*_ and *Srebf*_*1*_. Moreover, in a co-culture of 3T3-L1 adipocytes and RAW 264 macrophages, treatment with GA resulted in a significant reduction in the expression of TNF, CCl_2_ and NOS_2_. Consequently, GA may be mitigate adipose tissue inflammation by suppressing the expression of inflammatory mediators, either within the white adipose tissue or in macrophages (Tanaka et al., 2020b).

Secondly, GA treatment can enhance lipid metabolism by improving mitochondrial function. Mitochondria are crucial for cellular energy production and lipid metabolism (Pernas, 2024). Moreover, GA can promote lipid metabolism by improving mitochondrial function, a key factor in cellular energy production and lipid metabolism. Specifically, GA significantly increased the expression of PGC1α target genes such as mitochondrial transcription factor A, nuclear respiratory factor-1, and nuclear respiratory factor-2, thereby upregulating fatty acid β-oxidation enzymes like carnitine palmitoyltransferase-I, and promoting fatty acid oxidative metabolism in mitochondria (Doan et al., 2015). Furthermore, in rats induced with HFD, GA reduced body weight, adipose tissue weight (peritoneal and epididymal), triglyceride (TAG) level, low-density lipoprotein cholesterol level, phospholipids, and total cholesterol level by enhancing antioxidant activity (Hsu and Yen, 2007). For example, in db/db mice fed with a high-fiber diet, GA enhanced intracellular antioxidant defenses, inhibited reactive oxygen species (ROS) production, and boosted hepatic antioxidant enzymes (GSH, SOD, GST), thereby promoting lipid metabolism (Hsu and Yen, 2007; Lee et al., 2021; Punithavathi V. et al., 2011).

Thirdly, GA improved lipid metabolism by increasing thermogenesis, which was associated with the AMPK/Sirt1/PGC1α pathway. Brown adipose tissue (BAT) is integral to lipid metabolism as it increased heat production through enhanced energy metabolism (Jung et al., 2019). Bioinformatics analyses have identified SIRT1 as a significant player in thermogenesis. PGC-1α is known to regulate thermogenesis in BAT (Rao et al., 2014; Zhang J. et al., 2022). In the BAT of HFD-fed mice, treatment with GA elevated the expression of SIRT1 and PGC1-alpha mRNA, enhancing thermogenesis and improving overall body metabolism (Paraíso et al., 2019). Furthermore, GA regulated key thermogenic factors primarily by activating the AMPK pathway. GA treatment activated this pathway and significantly upregulated the expression of UCP1, a crucial regulator of heat production in BAT. It also increased the expression of energy expenditure-related genes such as UCP3, PGC1β, and β3-Adr, contributing to body weight reduction in HFD-induced obese mice (Doan et al., 2015).

In conclusion, GA significantly promoted lipid metabolism both directly, by enhancing fatty acid and triglyceride metabolism, and indirectly, through the inhibition of inflammation, improvement of mitochondrial function, and stimulation of thermogenesis.

#### 3.1.3 Inducing apoptosis of adipocytes

GA has been demonstrated to reduce lipid accumulation by inducing adipocyte apoptosis, a process that entails the regulated, active death of adipocytes through apoptosis pathways. GA triggers adipocyte apoptosis via two primary pathways, outlined as follows:

GA can induce adipocyte apoptosis through the intrinsic (mitochondrial) pathway. In 3T3-L1 preadipocytes, GA increased the expression and release of mitochondrial cytochrome c into the cytoplasm, which significantly upregulated the expression of apoptosis-related enzymes, such as caspase-3 and caspase-9, thereby reducing cell viability (Hsu F.-L. et al., 2007). Additionally, in subcutaneous preadipocytes, GA altered the expression ratio of pro- and anti-apoptotic members of the Bcl-2 family, decreasing anti-apoptotic proteins like Bcl-2 and Bcl-XL, and markedly increasing pro-apoptotic proteins such as Bax, Bad, and Bak, which reduces the viability of these cells (Jin et al., 2022). Indeed, the extrinsic apoptosis pathway, activated by death receptors, is crucial for cellular apoptosis. This pathway is primarily mediated by fas cell surface death receptor/fas ligand (Fas/FasL) and significantly modulated by the p53 protein (Mustafa et al., 2021; R. Zhao et al., 2020). Similarly, GA modulated the Fas/FasL pathway, contributing to p53-mediated induction of apoptosis in adipocytes. In 3T3-L1 preadipocytes, GA elevated the expressions of Fas, FasL, and p53, diminished adipocyte viability, and decreased lipid accumulation (Hsu C.-L. et al., 2007).In summary, GA disrupted adipocyte viability and induces apoptosis via both the mitochondrial and death receptor pathways.

#### 3.1.4 Promoting adipocyte differentiation

GA indirectly regulated lipid levels by promoting adipocyte differentiation. Torres et al. have found that this is a process that increases the production of adipokines (such as lipocalin) and enhances oxidase activity (Pérez-Torres et al., 2021). A key factor in this process is PPARγ, a ligand-activated transcription factor from the nuclear receptor family, pivotal in adipocyte differentiation (Hernandez-Quiles et al., 2021). In diet-induced obese mice, GA reduced serum triglyceride concentrations and significantly increased PPARγ protein level in white adipose tissue, thereby enhancing adipocyte differentiation (Bak et al., 2013). Additionally, in GA-treated mouse 3T3-L1 cells, expressions and levels of both adiponectin, a crucial protein hormone for this process—and fatty acid-binding protein-4 (Fabp4), a target of PPARγ and a marker of adipocyte differentiation were increased. Furthermore, GA treatment boosted the secretion and expression of lipocalin, reduced adipocyte viability, and ameliorated lipid accumulation (Makihara et al., 2016; Tanaka et al., 2020b).

In conclusion, it was evident that GA promoted adipocyte differentiation, leading to enhanced production of adipokine, which in turn indirectly influenced lipid levels.

### 3.2 Anti-AS effects of GA

**FIGURE 2:**
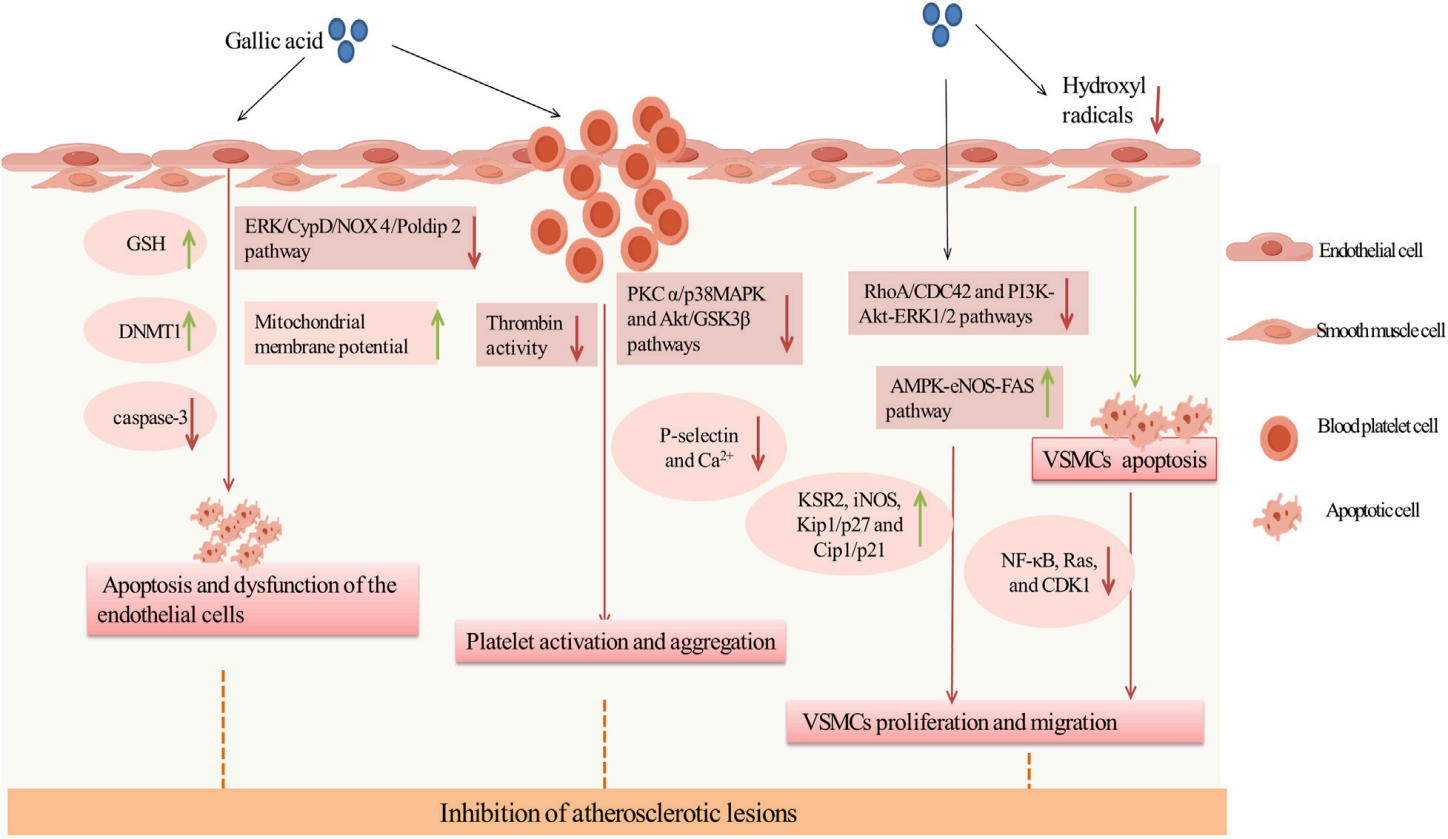
Inhibition of atherosclerotic lesions by GA. Green arrows and red arrows indicate promotion and inhibition, respectively. Description: This figure illustrates how GA inhibits atherosclerotic lesions through multiple mechanisms. GA protects endothelial cells by increasing GSH levels and DNMT1 expression to reduce apoptosis, while improving mitochondrial function by inhibiting the ERK/CypD/NOX4 pathway. It also inhibits platelet activation and aggregation by suppressing thrombin activity, downregulating the PKC and p38/MAPK pathways, reducing Ca^2^⁺ influx, and decreasing P-selectin expression. Additionally, GA suppresses VSMC proliferation and migration by inhibiting the RhoA/CDC42 and PI3K-Akt-ERK1/2 pathways, while promoting VSMC apoptosis through the AMPK-eNOS-FAS pathway and mitigating oxidative stress caused by hydroxyl radicals (•OH). These effects collectively stabilize atherosclerotic plaques and prevent their progression. Green arrows indicate promotion, while red arrows represent inhibition.

AS is a chronic inflammatory vascular disease and the basis of ASCVD (Hafiane and Daskalopoulou, 2022). The pathology of atherosclerosis involves damage to arterial endothelial cells, typically caused by factors such as hypertension, high cholesterol, and diabetes. This damage leads to the oxidation of LDL within the vessel walls, initiating an inflammatory response and the formation of foam cells. These foam cells gradually develop into plaques. As these plaques grow, they cause significant changes in vascular function and structure, including platelet activation, proliferation and migration of vascular smooth muscle, and thickening and hardening of the arterial walls with narrowing of the lumen (Jiang et al., 2022). These changes decrease blood flow, increase the risk of thrombosis, and can ultimately lead to various cardiovascular events such as myocardial infarction, stroke, and peripheral arterial disease (Dasagrandhi et al., 2022). Studies have demonstrated that GA exhibited multiple biological activities and showed significant potential in the treatment of AS. GA enhanced endothelial cell viability through its antioxidant properties and by regulating apoptosis-related enzymes. It also reduced platelet activation and aggregation by inhibiting P-selectin on platelets. Additionally, GA effectively regulated the proliferation and migration of VSMCs by modulating key cell cycle signals and pathways (Appeldoorn et al., 2005; Chang et al., 2012a; Chung et al., 2020). In conclusion, GA could intervene in the pathogenesis of AS through multiple mechanisms, slowing disease progression and improving prognosis, making it a promising candidate for AS treatment (Figure 2).

#### 3.2.1 Inhibiting apoptosis and dysfunction of endothelial cells

Research showed that GA preserved vascular health by preventing endothelial cell apoptosis and dysfunction, making it a key strategy against AS. Initially, GA can directly reduce apoptosis. In various mouse models, DNA (Cytosine-5)-Methyltransferase 1 (DNMT1) was crucial for cell survival, and proteasome inhibitors can prevent endothelial cell apoptosis and reduce mortality, significantly aiding in endothelial protection (Wesley et al., 2024; Zhao et al., 2024). In EAhy926 and HBEC-5i cells exposed to a mixture of homocysteine, adenosine, and tumor necrosis factor (TNF) (DL Hcy Ado TNF), GA effectively restored DNMT1 expression, reduced the activity of the chymotrypsin-like proteasome, significantly inhibited caspase-3 expression, and alleviated apoptotic effects and granule formation (Kam et al., 2014).

Secondly, GA can damage endothelial cells by reducing oxidative stress. Endothelial dysfunction, a precursor in atherosclerosis pathogenesis, was primarily driven by oxidative stress (Panda et al., 2022). GA acted as an antioxidant to counteract oxidants generated in the peroxidase cycle. In human microvascular endothelial cells (HMEC-1) exposed to high concentrations of hydrogen peroxide, GA reduced oxidants produced in the peroxidase cycle, and significantly enhanced cell viability (Serrano et al., 2010). Furthermore, in a cultured human umbilical vein endothelial cell model of oxidative stress (o-toluene-3-phenol), GA at relatively low concentrations enhanced endothelial cell viability and significantly reduced o-toluene-3-phenol-induced cytotoxicity. This was primarily attributable to an elevation in total intracellular GSH levels in the presence of low concentrations of GA. However, no enhancement in SOD or catalase activity was observed (Goszcz et al., 2017). In addition, recent studies have shown that GA can also ameliorate endothelial cell injury by inhibiting endothelial cell mitochondrial dysfunction, mainly through the ERK/CypD/NOX 4/Poldip 2 pathway. In Ang II-induced HUVECs, GA inhibited Extracellular Signal-Regulated Kinase (ERK) phosphorylation, Cyclophilin D (CypD) expression, the interaction of NOX4 and Poldip 2, and lowered mitochondrial ROS levels, resulting in an increase in the mitochondrial membrane potential of the HUVECs cells, thereby alleviating endothelial mitochondrial dysfunction (Sun et al., 2023). Thus, GA may inhibit oxidative stress and ameliorate endothelial cell dysfunction by increasing total GSH levels and mitochondrial membrane potential.

#### 3.2.2 Inhibiting platelet activation and aggregation

Inhibition of platelet activation and aggregation constituted a key anti-atherosclerosis mechanism of GA in the treatment of cardiovascular diseases.This effect was primarily associated with platelet P-selectin. In both normolipidemic C57/Bl6 and aged atherosclerotic ApoE-deficient mice, GA improved vascular cell adhesion molecule function and inhibit platelet activation prior to platelet activation. Further research has found that GA inhibited the interaction between P-selectin and platelets in HL60 cells, thereby preventing platelet activation (Appeldoorn et al., 2005). Futhermore, in platelet-rich plasma stimulated by ADP or U46619 (a thromboxane A2 analog), GA reduced the adherence of platelets to white blood cells and inhibited the formation of platelet-leukocyte aggregates. It was shown that GA reduced intracellular Ca^2+^ level by inhibiting phosphorylation of Protein Kinase C alpha (PKCα) and p38 mitogen-activated protein kinase (p38 MAPK) in platelets, as well as phosphorylation of Akt and glycogen synthase kinase 3 beta (GSK3β), leading to a significant reduction in ADP- and U46619-induced platelet aggregation and platelet P-selectin expression in a concentration-dependent manner (Chang et al., 2012b). In conclusion, GA improves platelet aggregation by inhibiting the expression of P-selectin, which may be related to PKC α/p38 MAPK and Akt/GSK3β pathways.

Additionally, GA could reduce the initial activation of platelets by inhibiting thrombin activity. Negrier et al. identified thrombin as a key regulatory factor in platelet activation and aggregation, enhancing adhesion and aggregation between platelets by promoting fibrin formation (Negrier et al., 2019). In platelet aggregation assays involving thrombin-stimulated platelets, GA demonstrated a reduction in aggregation by approximately 35%. Subsequent studies confirmed that GA significantly inhibited thrombin by directly binding to the thrombin protein, thereby rapidly stabilizing its conformation and reducing platelet aggregation (Zhang et al., 2022b).

In summary, GA reduces platelet activation and aggregation by inhibiting P-selectin and thrombin activity.

#### 3.2.3 Inhibiting proliferation and migration of VSMCs

**FIGURE 3:**
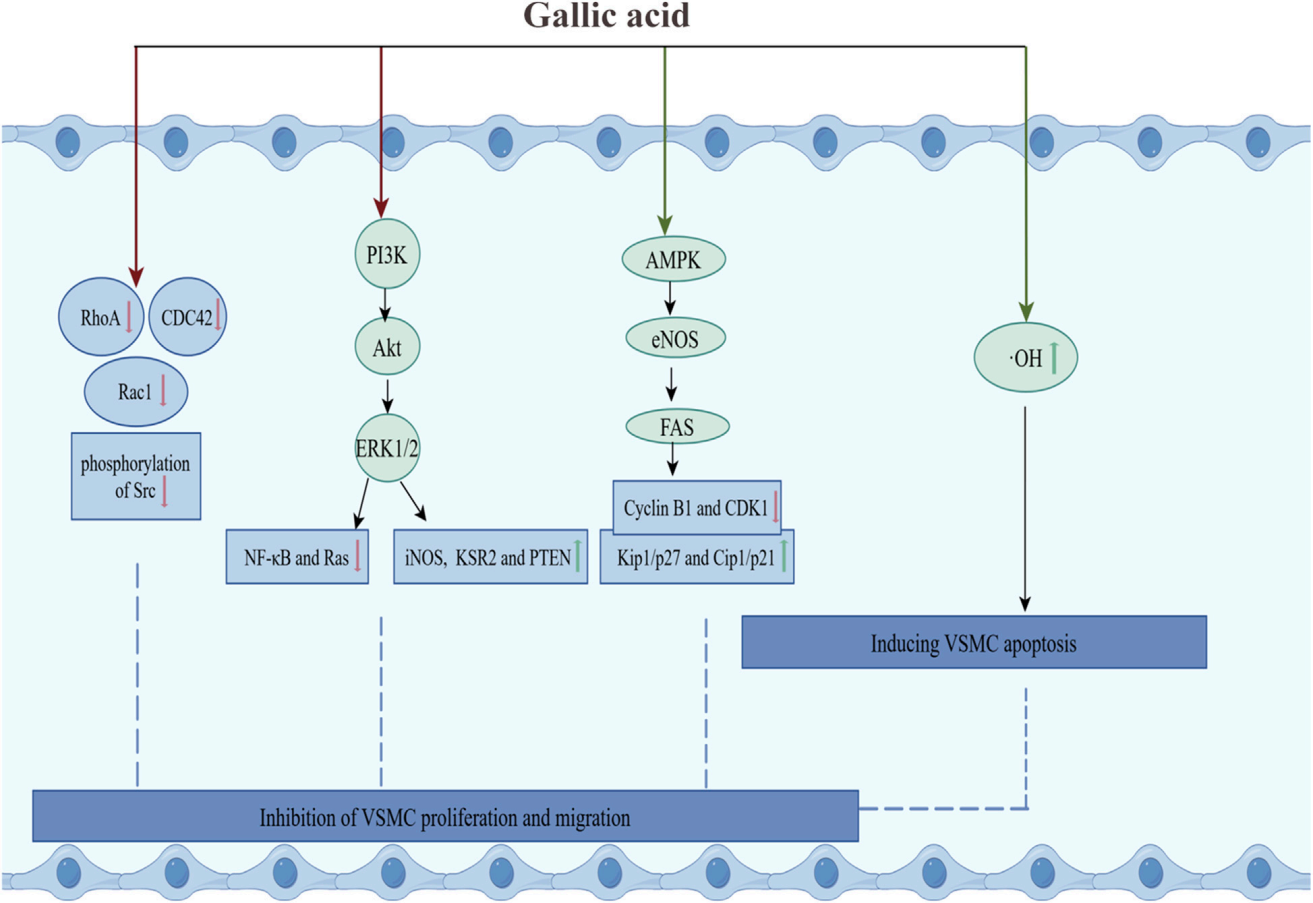
GA inhibited the proliferation and migration of VSMCs. Green arrows and red arrows indicate promotion and inhibition, respectively. Description: This figure illustrates how GA inhibits VSMC proliferation and migration while inducing apoptosis. GA suppresses the RhoA/CDC42 and PI3K-Akt-ERK1/2 pathways, reduces the activation of NF-κB and Ras, and increases the expression of iNOS and KSR2. Additionally, GA promotes the AMPK-eNOS-FAS pathway, enhances Kip1/p27 and Cip1/p21 levels, and inhibits the expression of cyclin B1 and CDK1. Moreover, GA reduces hydroxyl radicals and alleviates oxidative stress to induce apoptosis. Green arrows indicate promoting effects, while red arrows represent inhibitory effects.

Firstly, GA inhibited the proliferation of VSMCs by inducing their apoptosis. In cultured VSMCs from rat aortas, GA treatment inhibited oxidative stress caused by hydroxyl radicals and promoted apoptosis, characterized by cytoplasmic shrinkage, vesicle formation, and nuclear condensation, thereby inhibiting the proliferation of vascular smooth muscle cells (Qiu et al., 2000). Secondly, Clark et al. have demonstrated that GA decelerated cell cycle progression in VSMCs through reducing signaling pathways associated with growth, mobility, and senescence, thereby inhibiting the over-proliferation of VSMCs (Clark et al., 2022). In TNF-α-stimulated A7r5 rat aortic VSMC, GA inhibited the expression and phosphorylation of the cytoskeletal proteins ras homolog family member A (RhoA), Ras-related C3 botulinum toxin substrate 1 (Rac1), and cell division cycle 42 (CDC42). This resulted in a reduction in TNF-α-induced cell migration. Furthermore, GA inhibited the phosphorylation of PI3K, Akt and ERK, and regulated the expression of inflammatory proteins (reducing the activation of NF-κB and Ras, and increasing the expression of iNOS and Kinase Suppressor of Ras 2), and increased the expression of Phosphatase and Tensin Homolog deleted on Chromosome 10 (PTEN), thereby inhibiting TNF-α-induced cell proliferation (Chung et al., 2020). Furthermore, in OA-treated VSMC, GA treatment activated AMPK and eNOS, and inhibited fatty acid synthase (FAS), decreased the levels of cytokinin B1 and cytokinin-dependent kinase 1 (CDK1), and increased the levels of Kip1/p27 and Cip1/p21, which led to the accumulation of the G2/M phase of the cell cycle, thereby slowing down cell cycle progression and inhibiting VSMC proliferation. The specific inhibitor of AMPK, Compound C, was observed to reduce GA-induced eNOS activation and nitric oxide production, thereby underscoring the pivotal role of AMPK in this process (Ou et al., 2013). In summary, GA inhibited the RhoA/CDC42 and PI3K-Akt-ERK1/2 pathways and activated the AMPK-eNOS-FAS signaling pathway, thereby suppressing the migration and proliferation of VSMCs (Figure 3).

### 3.3 Anti-arterial stiffness effect of GA

Arterial stiffness refers to the loss of elasticity and hardening of the arteries, typically measured by pulse wave velocity (PWV) (Aimagambetova et al., 2024). Vascular stiffening is a complex process driven by multiple factors, including vascular calcification, elastic fiber degradation, and collagen deposition (Ribeiro-Silva et al., 2021). It is not only a consequence of AS but also an important risk factor and a predictor of ASCVD (Szołtysek-Bołdys et al., 2024).

Recent studies demonstrated that GA exhibited significant vascular protective effects and alleviated arterial stiffness. Firstly, GA inhibited vascular calcification by blocking the BMP2-Smad1/5/8 signaling pathway.In vascular smooth muscle cells (VSMCs) induced by inorganic phosphate (Pi), GA inhibited vascular calcification by interfering with the osteogenic signaling pathway through suppressing BMP2 upregulation and Smad1/5/8 phosphorylation (Kee et al., 2014). Secondly, GA inhibited arterial stiffness by preventing the degradation of elastic fibers and suppressing collagen deposition. Studies have shown that matrix metalloproteinases (MMPs) degrade elastic fibers, while transforming growth factor-β (TGF-β) induces collagen deposition by activating fibrosis-related genes (Giachelli et al., 2024; Vallée and Lecarpentier, 2019). In rats induced by advanced glycation end products (AGEs), GA downregulated expressions of MMP-2, MMP-9, and TGF-β, thereby inhibiting extracellular matrix (ECM) remodeling and calcification and protecting vascular elasticity. Furthermore, additional studies found that this effect was associated with GA’s ability to inhibit ROS generation, as well as the expression of NF-κB and the level of TNF-α, which was further validated in AGEs-induced H9C2(2–1) (Umadevi et al., 2014; 2013). In conclusion, GA mitigated arterial stiffness by inhibiting vascular calcification through the suppression of the BMP2-Smad1/5/8 signaling pathway and protecting elastic fibers as well as reducing collagen deposition via its antioxidant and anti-inflammatory effects.

## 4 Therapeutic potentials of GA against the drug-controllable risk factors of ASCVD

Hyperglycemia and hypertension are key modifiable risk factors in the prevention and management of ASCVD. These metabolic disorders not only accelerate arterial hardening and damage to the vascular walls but also significantly increase the incidence of cardiovascular events. Borghi et al. have found that comprehensive control of these factors, through lifestyle changes or pharmacological interventions, can significantly reduce the incidence and mortality rates of ASCVD (Borghi et al., 2022; Haas and McDonnell, 2018). Traditional medications are primary interventions but often have adverse side effects, driving the search for natural product-based alternatives. Indeed, GA improves glucose metabolism, which is beneficial for controlling high blood sugar. Specifically, GA enhances insulin sensitivity by regulating the signaling pathways involved in glucose uptake and utilization. This not only helps in controlling blood sugar levels but also addresses insulin resistance (Sousa et al., 2020; Zhang et al., 2024). Also, it impairs vasodilatory function through oxidative stress and inflammation, reducing blood pressure level.Therefore, by targeting fundamental risk factors such as hypertension and diabetes, GA not only addresses the root cause of ASCVD deterioration but also provides diverse disease management strategies.

### 4.1 Hypoglycemic effect of GA

**FIGURE 4:**
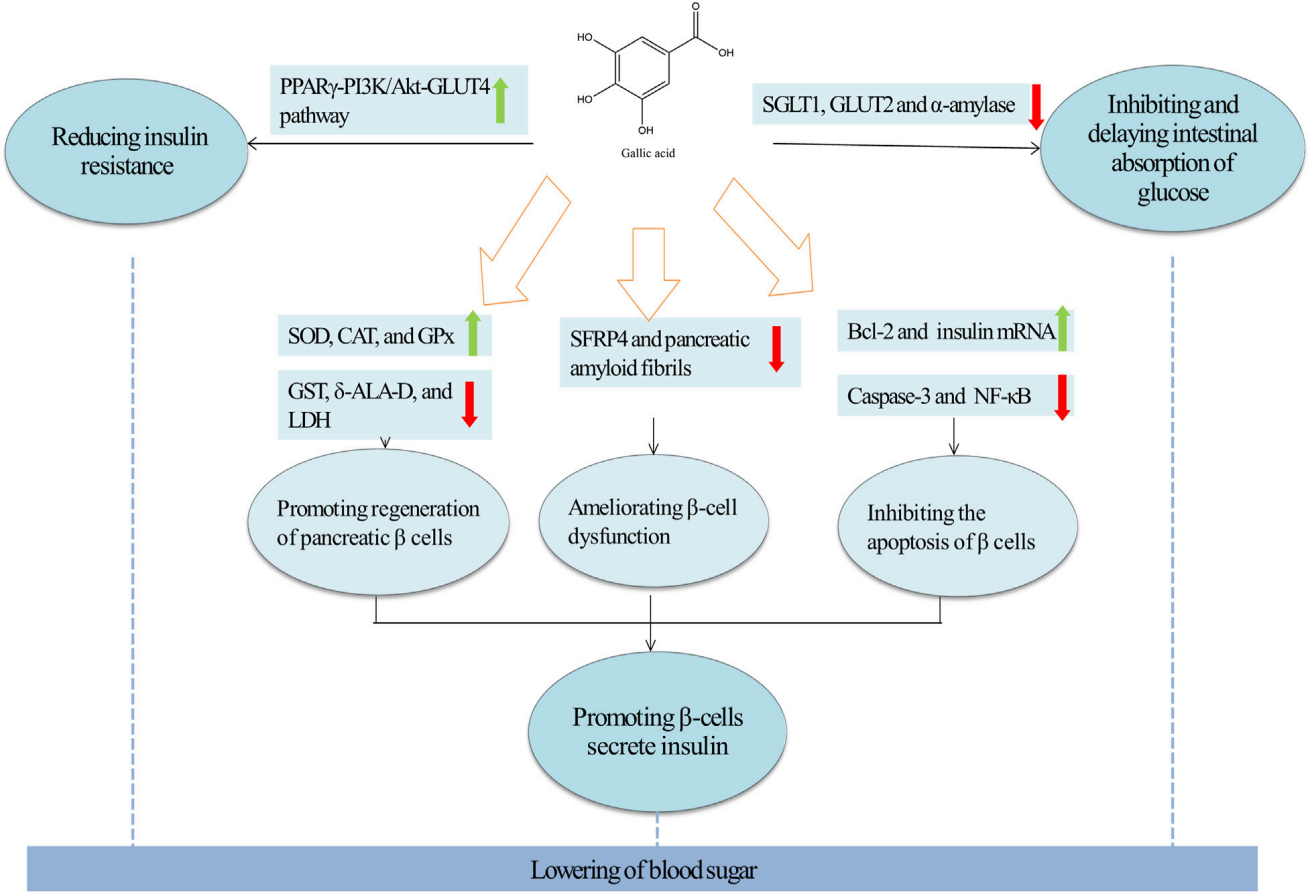
Molecular targets and mechanism of action of GA in T2DM. Green arrows and red arrows indicated promotion and inhibition, respectively. Description: This figure illustrates the molecular mechanisms by which GAexerts therapeutic effects in T2DM. GA enhances insulin secretion by inhibiting oxidative stress, apoptosis, and the formation of SFRP4. Simultaneously, GA improves insulin resistance by activating the PPARγ-PI3K/Akt-GLUT4 signaling pathway, thereby increasing glucose uptake. Additionally, GA delays glucose absorption by inhibiting the activities of SGLT1, GLUT2, and α-amylase. In the figure, green arrows indicate processes or pathways promoted by GA, while red arrows highlight inhibitory effects.

Researches indicate that the onset of type 2 diabetes (T2DM) involves significant islet dysfunction, β cell failure, and the emergence of insulin resistance (Burillo et al., 2021; Lawlor et al., 2017); Consequently, enhancing the quantity of pancreatic islet β cells and reducing insulin resistance are crucial in diabetes management (Prasad et al., 2023). GA, recognized for its hypoglycemic properties, plays a beneficial role in both the prevention and treatment of diabetes (Xu et al., 2021). The efficacy of GA is largely due to its ability to stimulate insulin secretion from β cells, reduce insulin resistance, and inhibit the intestinal absorption of glucose (Figure 4).

#### 4.1.1 Promoting insulin secretion of β-cells

Gerst et al. identified that mitigating β cell damage and fostering β cell regeneration significantly boosts insulin secretion from these cells (Gerst et al., 2021; Rady et al., 2022).Thus, strategies to restore β cell functionality typically involved promoting cell proliferation and minimizing damage (Lewis and Wells, 2021; Lu et al., 2020; Tomita, 2017). Researches have demonstrated that GA primarily enhanced regeneration of β cells and reduced β cell damage, thereby augmenting insulin secretion. The detailed mechanisms are elaborated below.

Initially, GA promoted the regeneration of pancreatic β cells and enhance insulin secretion. In diabetes rats, GA treatment resulted in a dose-dependent decrease in blood glucose level, as evidenced by lowered AUC_glucose_, insulin levels, and Homeostasis Model Assessment of Insulin Resistance (HOMA-IR) indices (Abdel-Moneim et al., 2017; Variya et al., 2020). Additionally, in male Wistar rats with diabetes induced by streptozotocin (STZ), GA improved pathological alterations in pancreatic islet cells, promoted β-cell regeneration, and boosted insulin output. From a mechanistic standpoint, GA promoted pancreatic β-cell regeneration through its antioxidant properties (Latha and Daisy, 2011). In STZ-induced diabetic male albino rats, GA enhanced antioxidant capacity by altering biochemical conditions in pancreatic tissues. It decreased the activity of pancreatic peroxidases, purinergic enzymes, detoxifying enzymes (GST), heme biosynthesizing enzymes (δ-ALA-D), and glycolysis enzymes (LDH), which led to reduced level of blood glucose and decreased pancreatic weight (Kade et al., 2014). Furthermore, in a rat model of STZ-induced diabetes, GA significantly reduced thiobarbituric acid-reactive substances and lipid hydroperoxides, enhanced activities of superoxide dismutase (SOD), catalase (CAT), and glutathione peroxidase (GPx). Histopathological analysis showed that GA restored pancreatic beta cells and increased insulin secretion (Punithavathi V. R. et al., 2011).

Secondly, GA enhanced insulin secretion by ameliorating damage of β-cells. Recent studies have demonstrated that secreted frizzled-related protein 4 (SFRP4) is a potential biomarker for β-cell dysfunction in type II diabetes, associated with reduced insulin secretion (Bukhari et al., 2019; Zhang et al., 2020). In diabetic mice induced by a high-calorie diet, oral administration of GA significantly lowered serum SFRP4 level, which led to reductions in body weight and blood glucose level (Bukhari et al., 2022). Furthermore, in RINm5F β cells exposed to high glucose, palmitate esters, or both, GA enhanced cellular survival pathways by upregulating Bcl-2 activity, downregulating caspase-3 activity, reducing DNA damage, weakening nuclear factor kappa-light-chain-enhancer of activated B cells (NF-κB) signaling, and increasing the expression of insulin mRNA, thereby promoting insulin secretion (Sameermahmood et al., 2010). Fibrin deposition (also known as islet amyloid polypeptide) has been identified as one of the factors leading to the death of islet β cells (Salazar Vazquez et al., 2020). Also, GA prevented the formation of pancreatic amyloid fibrils, thereby protecting pancreatic β cells and enhancing insulin secretion. *In vitro*, GA could interacted with natural insulin, inhibiting the key nucleation process essential for the growth of fibrils, thus hindering the formation of amyloid fibers and safeguarding the insulin structure and curtailing protofibril formation (Jayamani and Shanmugam, 2014).

#### 4.1.2 Reducing insulin resistance

Caturano et al. showed that addressing insulin resistance could significantly improve blood glucose control and was a feasible strategy for diabetes management (Caturano et al., 2024; Wang et al., 2019). In streptozotocin-induced experimental type 2 diabetic rats fed a high-fat diet, GA significantly reduced plasma insulin and HOMA-IR levels and normalised changes in insulin resistance levels, thereby reducing body weight and fasting blood glucose (Gandhi et al., 2014). Therefore, GA can lower blood glucose by alleviating insulin resistance GA attenuated insulin resistance primarily by activating the protein kinase B (Akt) signalling pathway. In fructose-induced diabetic rats, GA increased the expression of proteins related to hepatic insulin signalling, including insulin receptor (IR), insulin receptor substrate 1 (IRS-1), phosphoinositide 3-kinase (PI3K), Akt and glucose transporter 2 (GLUT2) (Huang et al., 2016). Furthermore, in experimental type 2 diabetic rats induced by a high-fat diet and streptozotocin, GA treatment lowered the levels of glucose-6-phosphatase and fructose-1,6-bisphosphatase, facilitating glucose absorption. This was associated with a significant increase in the expression of peroxisome proliferator-activated receptor gamma (PPARγ) mRNA. Molecular docking studies showed that PPARγ interacted well with GLUT4, GLUT1, PI3K and p-Akt, indicating that GA could attenuate insulin resistance by activating the PPARγ-PI3K/p-Akt -GLUT4 signalling pathway (Gandhi et al., 2014; Huang et al., 2018; Variya et al., 2020). It was noteworthy that in fructose-induced diabetic rat model, treatment with GA increased expressions of Glut4, PPAR-γ and pAkt proteins, but not phosphorylated AMP-activated protein kinase (pAMPK) in the epithelial white adipose tissue, suggesting that GA attenuated insulin resistance through the Akt signalling pathway (Variya et al., 2020). However, pAMPK was not restored, which further suggested that GA attenuated insulin resistance through the Akt signalling pathway.

#### 4.1.3 Inhibiting and delaying intestinal absorption of glucose

Numerous studies have demonstrated that type 2 diabetes could be therapeutically managed by reducing intestinal glucose absorption, primarily through the inhibition of intestinal carbohydrate hydrolases such as α-amylase, and sodium-dependent glucose transporter protein 1 (SGLT1) (Cefalo et al., 2019; Gong et al., 2020). GA has been shown to be effective in inhibiting and delaying intestinal absorption of glucose. In diabetic albino rats, GA treatment significantly decreased α-amylase activity, thereby disrupting the hydrolytic absorption of starch-like compounds, which led to a marked reduction *in situ* intestinal glucose absorption (Abdel-Moneim et al., 2022). In addition, in a glucose transport assay using Caco-2 cell monolayers, GA demonstrated specific inhibitory effects on SGLT1, reducing intestinal glucose absorption primarily by suppressing the transport of low concentrations of glucose (5 mM). This effect was further validated in a 2DG transport assay (Wang et al., 2021). In conclusion, GA effectively reduced *in situ* intestinal glucose absorption, primarily by suppressing the activity of the intestinal carbohydrate hydrolase α-amylase and by downregulating the levels of glucose transporter proteins in the gastrointestinal tract.

### 4.2 Hypotensive effect of GA

Hypertension is a cardiovascular disease that is characterized by persistently elevated arterial blood pressure. Blood pressure is usually represented by two numerical values, namely, systolic blood pressure and diastolic blood pressure (McEvoy et al., 2024). Studies have demonstrated that GA could ameliorate hypertension through multiple mechanisms, including reducing systolic blood pressure, decreasing the thickness and weight of the aortic wall, and mitigating cardiac fibrosis (Jin et al., 2017a; 2017b). The specific mechanism is as follows.

GA improved endothelial cell damage and reduced blood pressure by inhibiting activities of NO-related enzymes, such as the NADPH Oxidase (Nox) and endothelial nitric oxide synthase (eNOS). In spontaneously hypertensive rats (SHRs), GA inhibited components of the renin angiotensin II system and lowered systolic blood pressure. In addition, GA relaxed blood vessels and also reduced the thickness and weight of the aortic wall, thereby regulating blood pressure in the vascular system. This may be related to the inhibition of Nox and malondialdehyde (MDA) levels in cardiac tissue by GA. Furthermore, this was confirmed in Ang II-induced H9c2 cells (Jin et al., 2017b). Lind et al. discovered that eNOS was an enzyme responsible for producing NO, and was crucial for cardiovascular health and blood pressure regulation (Lind et al., 2017). In Ang II-induced C57BL/6J mice, GA inhibited immunoproteasome, trypsin, and chymotrypsin activities, thereby increasing eNOS degradation and NO levels. To verifiy the blood pressure-lowering effect of the eNOS/NO pathway, a specific inhibitor Nω-Nitro-L-arginine methyl ester (L-NAME) was used to block eNOS activity, which significantly eliminated the GA-mediated beneficial effects, such as the reduction in SBP, aortic thickening, and collagen deposition (Yan et al., 2020). In SHRs, GA significantly reduced both SBP and DBP. Subsequent studies demonstrated that pretreatment with the eNOS inhibitor L-NAME resulted in a reduction in NO production in human umbilical vein endothelial cells (HUVEC). GA treatment resulted in increased phosphorylation of eNOS and Akt, which subsequently induced NO production and inhibited angiotensin I converting enzyme (Kang et al., 2015). Therefore, GA reduced blood pressure by activating the eNOS/NO pathway, which may be related to the Akt pathway. Furthermore, studies have found that HDAC (histone deacetylase), epigenetic regulators that remove acetyl groups from histones, are closely associated with endothelial dysfunction, inflammation, and myocardial fibrosis through their epigenetic regulatory actions (Bahl and Seto, 2021). In NG-nitro-L-arginine methyl ester-induced hypertensive mice, GA significantly inhibited the expression of histone deacetylase 1 (HDAC1), histone deacetylase 1 (HDAC2) and atrial natriuretic peptide, which reduced SBP levels in chronic L-NAME-induced hypertensive mice, LV (left ventricle) posterior wall, septum thickness and cardiac fibrosis (Jin et al., 2017a). Additionally, recent studies demonstrated that GA was an effective dietary HDAC inhibitor with strong inhibitory effects on the activity of HDAC8 and class IIa/b HDACs. These findings suggested that GA exhibited antihypertensive and antifibrotic effects, highlighting its significant potential for clinical translation (Choi et al., 2018). In summary, GA alleviated symptoms of hypertension by increasing the level of NO.

Additionally, GA treated hypertension by inhibiting the expression of calcium/calmodulin-dependent protein kinase II (CaMKII), which was related to apoptosis. CaMKII is a key protein kinase that regulates the contraction and relaxation processes of cardiomyocytes (Carlson et al., 2022; Zhang et al., 2022c). In SHRs, GA significantly reduced the expression of four CaMKII isoforms: α, β, δ, γ, and the expressions of caspase-3, Bax, p53, and p300 proteins, thereby reducing angiotensin II-induced angiotensin II-induced apoptosis (Jin et al., 2018).

Overall, GA lowered blood pressure mainly by ameliorating endothelial cell damage and inhibiting apoptosis.

## 5 Anti-ASCVD effect of GA

Atherosclerosis contributes to ASCVD by narrowing arteries or forming thrombi, thereby obstructing blood flow to the heart (coronary artery disease), brain (ischemic stroke), or lower limbs (peripheral vascular disease). The main areas affected by ASCVD are 1) CHD, 2) cerebrovascular disease, and 3) peripheral vascular disease (Tannu et al., 2024). Studies showed that GA, due to its antioxidant properties, helped prevent and treat CHD and cerebrovascular diseases, contributing to the management of ASCVD. Specifically, GA reduced serum the levels of LDH, creatine phosphokinase (CPK), and creatine kinase-MB (CK-MB), regulated hemodynamic parameters, and offered protection against CHD (Ramezani-Aliakbari et al., 2017; Souri et al., 2023; Yang et al., 2023). Moreover, GA enhanced neuroprotective proteins, improved cerebral blood flow, and lowered blood-brain barrier permeability, providing potential therapeutic benefits for ischemic brain diseases (Praveen Kumar et al., 2021). Consequently, GA emerges as a promising drug for treating ASCVD.

### 5.1 Anti-CHD effects of GA

CHD, caused by coronary atherosclerosis, leads to myocardial ischemia and hypoxia due to lipid metabolism disorders, oxidative stress, and inflammation (Donia and Khamis, 2021; Han et al., 2024). Berezin et al. emphasize the importance of cardiac markers and function indices for diagnosing myocardial ischemia and infarction, with evaluations relying on cardiac hemodynamic and electrocardiogram parameters (Berezin and Berezin, 2020; Liu et al., 2022; Sarda and Thute, 2022). GA was a phenolic compound with a protective effect against CHD through its antioxidant properties. In ischemia-reperfused rat hearts, GA reduced histological damage in the heart, showing less edema, collagen fibers, and necrosis, and improved myocardial structure (Dianat et al., 2014). GA also lowered levels of serum cardiac markers like CK-MB and LDH, indicating cardioprotection (Shackebaei et al., 2022; Stanely Mainzen Prince et al., 2009). CK-MB is a specific marker for early myocardial injury, while LDH, a glycolytic enzyme, helps assess the extent of myocardial damage (Feng et al., 2024; Xu et al., 2024). Notably, in an isoproterenol (ISO)-induced ischemia/reperfusion (I/R) injury model, GA not only decreased the activity of cardiac biomarkers (CK-MB, CPK, and LDH) but also increased the activity of lysosomal enzymes such as β-glucuronidase and cathepsin D (Shackebaei et al., 2022), and in ISO induced MI in rats, GA also reduced serum cardiac troponin T (cTnT) level and the intensity of LDH-1 and LDH-2 isoenzyme bands (Priscilla and Prince, 2009).

Additionally, GA improved hemodynamic parameters, enhancing vasodilation, blood flow, and overall cardiac function. These changes collectively contributed to significant improvements in myocardial injury and infarction outcomes. In a rat model of four-vessel occlusion (4VO) I/R induced by PM, GA significantly enhanced cardiac function post-I/R by reducing hemodynamic parameters such as left ventricular developed pressure (LVDP), rates of pressure development (±dp/dt), and the rate-pressure product (RPP)(Radan et al., 2019). These findings demonstrated the effectiveness of GA in reducing myocardial cell damage (Dianat et al., 2014; Shackebaei et al., 2022; Stanely Mainzen Prince et al., 2009). This was linked to the antioxidant properties of GA, which specifically lowered cardiac Thiobarbituric Acid Reactive Substances (TBARS) level and enhanced the activity of antioxidant enzymes such as SOD, CAT, and GPX (Patel and Goyal, 2011; Ramezani-Aliakbari et al., 2017; Shackebaei et al., 2022). It is noteworthy that neutrophil activity plays a crucial role in improving the prognosis of myocardial infarction (Cristinziano et al., 2022). Calycosin and GA synergistically induced the expression of Leukotriene B4 12-Hydroxydehydrogenase (LTB4DH) in HepG2 cells and human neutrophils, which can be used to limit neutrophil infiltration and subsequent myocardial injury. Further confirmation was obtained in an isoproterenol-induced myocardial infarction mouse model, where these two LTB4DH inducers—namely calycosin and GA—significantly reduced the levels of myeloperoxidase (MPO) and MDA in cardiac tissues, thereby attenuating the cardiac morphological changes induced by isoproterenol (Cheng et al., 2015).

### 5.2 Anti-cerebral ischemic activity of GA

Cerebral ischemia, caused by a loss of blood supply, leads to harmful processes like glutamate excitotoxicity, calcium overload, oxidative stress, and inflammation, resulting in cell death (Kaur and Sharma, 2022; Suzuki et al., 2021). Effective treatment targets hypoxia/reoxygenation injury and aims to reduce inflammation and oxidative stress (Geng et al., 2020; Guo et al., 2019). In fact, GA offered potential benefits for ischemic brain diseases by inhibiting oxidative stress, inflammation, and apoptosis.

Firstly, as a natural antioxidant, GA has shown potential in the prevention and treatment of ischemic brain diseases. In rats with I/R induced by bilateral common carotid artery (BCCA) occlusion, GA enhanced passive avoidance memory and tail-flick latency, thereby improving outcomes in cerebral ischemia/reperfusion injury. This was primarily due to GA pretreatment enhancing antioxidant defenses, inhibiting the functions of neurotoxicity-related proteins (Bax, TNF-α and caspase-3), and improving cerebral I/R injury in rats, thereby exhibiting neuroprotective properties (Abdelsalam et al., 2023; Farbood et al., 2013; Nabavi et al., 2016; Praveen Kumar et al., 2021). GA alleviated behavioral and electrophysiological deficits induced by cerebral hypoperfusion ischemia (CHI) through its antioxidant and free radical scavenging properties, providing significant neuroprotection. For example, in a rat model of CHI induced by permanent bilateral common carotid artery occlusion (2VO), GA significantly restored spatial memory, increased time spent in the target quadrant, and improved memory consolidation (Sarkaki et al., 2014). It is noteworthy that cerebral ischemic injury can lead to vascular dementia (VD). Studies have found that GA also has therapeutic effects on VD. In a VD model induced by 2VO, GA demonstrated beneficial effects on 2VO-induced cognitive deficits. It significantly improved spatial memory in the Morris water maze by increasing non-enzymatic (total thiols) and GPx antioxidant levels (Korani et al., 2014).

Secondly, GA exerted neuroprotective effects against cerebral ischemia by reducing inflammation. In a rat model involving four-vessel occlusion (4VO)-induced I/R, GA significantly mitigated I/R-induced cognitive impairments and hippocampal long-term potentiation damage. This protective effect was associated with an increase in miR-146a expression and the anti-inflammatory cytokine IL-10, along with a reduction in the pro-inflammatory cytokine TNF-α levels (Bavarsad et al., 2023). Additionally, GA inhibited the levels of inflammatory mediators by activating microglia. In an experimental ischemic stroke model, GA treatment significantly reduced brain edema and infarct volume, thereby improving neurological function in Middle Cerebral Artery Occlusion (MCAO) mice, including motor skills, sensory sensitivity, balance, and reflexes. Furthermore, GA treatment decreased the levels of the microglial marker Iba-1 mRNA in the ipsilateral hemisphere, indicating microglial activation. Importantly, during the acute phase of ischemic injury, GA significantly reduced the levels of M1 markers (iNOS, COX-2, MCP-1) while increasing the levels of M2 markers (Arg-1, CD206, IL-10). This dual action not only inhibited the transition of microglia to the pro-inflammatory M1 subtype but also promoted their shift to the anti-inflammatory M2 subtype. As a result, GA significantly lowers the levels of inflammatory mediators (IL-1β, MCP-1, TNFα, IL-6, MIP-2) while the expression of tight junction proteins (ZO-1 and claudin) was increased (Qu et al., 2022).

Finally, GA alleviated cerebral ischemic injury by inhibiting apoptosis in brain microvascular endothelial cells. In Na₂S₂O₄-induced MCAO rats, GA significantly increased the expression of cytochrome C in mitochondria while reducing its expression in the cytoplasm. This led to a marked decrease in neurological deficit scores, total infarct volume, and TUNEL-positive cells in each infarct region. In Na₂S₂O₄-induced SH-SY5Y cells, GA inhibited mitochondrial permeability transition pore (MPTP) opening and prevented the dissipation of mitochondrial membrane potential, significantly increasing ATP levels. As a result, GA mitigated mitochondrial dysfunction, thereby protecting cells from apoptosis or necrosis (Sun et al., 2014). Further research revealed that GA inhibited the opening of MPTP by regulating the ERK-CypD axis, thereby preventing apoptosis. In BCCA-induced I/R rats, GA reduced neurological deficit scores and the percentage of cleaved-caspase-9 positive cells (relative to DAPI + cells). In H2O2-induced SH-SY5Y cells, GA treatment inhibited the binding of CypD to adenine nucleotide translocase and enhanced the phosphorylation of ERK, leading to reduced expression of CypD. This resulted in desensitization to MPTP-induced permeability transition, thereby suppressing the expression of mitochondrial apoptosis signals, including initiator Cyto C, mediator caspase-9, and effector caspase-3. Consequently, the apoptosis rate of SH-SY5Y cells was significantly reduced (Sun et al., 2017).

Collectively, GA possessed anti-hyperglycemic, anti-hyperlipidemic, anti-hypertensive, anti-atherosclerotic, anti-CHD, and anti-cerebral ischemia properties (Table 1). In Table 1 we list detail information, models, dosage and application.

**TABLE 1:** Pharmacological effects of GA on ASCVD.

Pharmacological properties		Detail information	Models	Dosage, methods of administration and treatment courses	Application	References
Therapeutic effects of GA	Anti-obesity and hypolipidemic effect	Reducing the expression of SREBP-1c, LXRα, ACCα, CD36 and FATP2	HepG2 cells	50–200 μM for 24 h	*In vitro*	Tanaka et al. (2020a)
		Raising ketone body levels and decreasing TG, cholesterol, and fatty acid levels	Mice with HFD-induced NAFLD	50 and 100 mg/kg/day, orally for 16 weeks	*In vivo*	Chao et al. (2020)
		Upregulating expressions of lipolysis-related genes such as ATGL and HSL and activating the metabolic regulator AMPKα	Bovine subcutaneous adipocytes	0, 50, 100, or 200 μM for 48 h	*In vitro*	Jin et al. (2022)
		Increasing the autophagosome formation	In the HepG2 cell line	50μM, treated for 12 h	*In vitro*	Doan et al. (2015)
		Suppressing the mRNA expression of IL6, NOS2, PTGS2, and ADGRE1	In co-culturing 3T3-L1 adipocytes with RAW 264.7 macrophages	270 μmol/L incubated for 4 h	*In vitro*	Tanaka et al. (2020b)
		Elevating the expression of SIRT1 and PGC1-alpha mRNA	HFD-fed mice	100 mg/kg/body weight, orally forthe 60 day	*In vivo*	Paraíso et al. (2019)
		Upregulating the expression of caspase-3, caspase-9 Bax, Bad, and Bak, and decreasing the expression of Bcl-2 and Bcl-XL	3T3-L1 preadipocytes	0–250 μM incubated for 24, 48, and 72 h	*In vitro*	Hsu et al. (2007b)
		Increasing expression and release of adiponectin and Fabp4, and reducing adipocyte viability	3T3-L1 cells	0 and 100 µM for 96 h	*In vitro*	Makihara et al. (2016)
	Anti-AS effect	Inhibiting the interaction between P-selectin and platelets	HL60	500 μmol/L,washed for 3 min	*In vitro*	Appeldoorn et al. (2005)
		Inhibiting intracellular Ca^2+^levels in platelet cells and regulating PKC α/P38MAPK and Akt/GSK3 β pathways	Platelet rich plasma induced by stimuli ADP or U46619	100 μM or 500 μM incubated for 3 min	*In vitro*	Chang et al. (2012b)
		Increasing PTEN, Ras 2 kinase inhibitor and PTEN expression, and inhibiting TNF- α Induced VSMC proliferation and Ras and RhoA expressions	TNF- α stimulated VSMCs of A7r5 rat aorta	0–100μM, treated for 24 h	*In vitro*	Chung et al. (2020)
		Restoring the expression of DNMT1 and significantly inhibiting the expression of caspase-3	DL Hcy Ado TNF induced human endothelial cells (EAhy926 and HBEC-5i cells)	10–100 μM,administered for 4h	*In vitro*	Kam et al. (2014)
		Inhibiting of oxidative substances produced in the peroxidase cycle	HMEC-1 cells exposed to H_2_O_2_	100nM-1 μM	*In vitro*	Serrano et al. (2010)
		Increasing intracellular total GSH	Human umbilical vein endothelial cells cultured under oxidative stress	10–100 µM,measured at 4, 8, 12, and 24 h	*In vitro*	Goszcz et al. (2017)
		Inhibiting ERK/CypD/NOX4/Poldip2 signaling pathway	HUVECs treated with Ang II	10 μM incubated into the cells 24 h	*In vitro*	Sun et al. (2023)
		Inhibiting thrombin induced platelet aggregation	Thrombin induced platelets	12.50, 25.00 and 50.00 μmol/L, run for 15 min	*In vitro*	Zhang et al. (2022a)
		Enhancing oxidative stress of reactive oxygen species · OH	VSMCs of rat aorta	0, 10, 50, or 100 μg/mL, incubated for up to 48 h	*In vitro*	Qiu et al. (2000)
		Reducing cyclin B1 and cyclin dependent kinase 1 (cdc2), increasing kip/p27 and cip1/p21	OA treated VSMCs	10–30μM, treated for 48 h	*In vitro*	Ou et al. (2013)
		Reducing LVDP, ±dp/dt, and RPP	I/R rat model	7.5, 15, 30 mg/kg, orally for 10 days	*In vivo*	Stanely Mainzen Prince et al. (2009)
	Anti-arterial stiffness effect	Inhibiting BMP_2_-Smad1/5/8 signaling pathway	Pi induced VSMCs	—	*In vitro*	Kee et al. (2014)
		Downregulating the levels of MMP-2, MMP-9, and TGF-β	AGEs induced rats	25 mg/kg/d administered from 30 d	*In vivo*	Umadevi et al. (2014)
Preventive effects of GA	Anti- diabetic effect	Decreasing the blood glucose levels, AUC (glucose), insulin levels, and HOMA-IR indices	db/db mice	100 mg/kg/day, orally for 42 days	*In vivo*	Latha and Daisy (2011)
		Increasing the activity of pancreatic peroxidases, purinergic enzymes, GST, δ-ALA-D and LDH	Male albino rats with diabetes induced by STZ	25 mg/kg/day, orally for35 days	*In vivo*	Kade et al. (2014)
		Increasing Bcl-2 activity and reducing caspase-3 activity, DNA damage and NF-κB signaling	RINm5F β cells exposed to high glucose, palmitate esters, or both	In the presence and absence, treated for 24 h	*In vitro*	Sameermahmood et al. (2010)
		Increasing in the expression of PPARγ mRNA and activating the PPARγ-PI3K/Akt-GLUT4 pathway	Type 2 diabetes rats induced by STZ fed with HFD	20 mg/kg/day, orally for 30 days	*In vivo*	Gandhi et al. (2014)
		Increasing the expression of IR, IRS-1, and GLUT2, and activating the PI3K/Akt pathway	HFD-induced diabetic rats	10 or 30 mg/kg/day, orally for the last 4 weeks	*In vivo*	Huang et al. (2016)
		Improving serum cholesterol and intrahepatic ketogenic levels and reducing levels of allantoin, urinary protein, glucose, AMP and alanine	STZ induced hyperglycemia in mice	50 and 100 mg/kg/day, orally for 16 weeks	*In vivo*	Chao et al. (2014b)
		Decreasing α-amylase activity	In experiments with diabetic albino rats	20 mg/kg/day, orally for 6 weeks	*In vivo*	Abdel-Moneim et al. (2022)
	Anti-hypertensive effect	Attenuating the activity of histone deacetylase 1 and histone deacetylase 2, and the expression of ANP	H9c2 cells induced by L-NAME	50 and 100 mg/kg per day, injected into 3–6 weeks	*In vitro*	Jin et al. (2017a)
		Reducing the expression of the immunoproteasome catalytic subunits β2i and β5i, chymotrypsin-like and trypsin-like activities of the proteasome, and maintaining NO levels	C57BL/6J mice induced by Ang II	5 or 20 mg/kg body weight daily, orally for 2 weeks	*In vivo*	Yan et al. (2020)
		Inhibition of RAAS activity and expression of GATA_4_ and Nkx_2-5_	SHR	320 mg per day, administered from 8 till 24 weeks	*In vivo*	Jin et al. (2017b)
		Reducing the expression of four CaMKII isoforms: α, β, δ, γ, and the expression of caspase-3, Bax, p53, and p300 proteins	SHR	1% in tap water, administered for 4 months	*In vivo*	Jin et al. (2018)
	Anti-CHD effect	Increasing the concentration of lysosomes β-glucuronidase and cathepsin D, decreasing the activity of LDH, CPK, and CK-MB	ISO male albino Wistar I/R rats	25 and 50 mg/kg, orally for 10 days	*In vivo*	Priscilla and Prince (2009)
		Reducing cTnT level and the intensity of LDH-1 and LDH-2 isoenzyme bands	ISO induced MI rats	15 mg/kg daily for a period of 10 days	*In vivo*	Badavi et al. (2014)
		Increasing of LTB4DH expression and metabolic conversion of LTB4, reducing LTB4 level and neutrophil survival	Rat I/R model	7.5, 15, 30 mg/kg, orally for 10 days	*In vivo*	Cheng et al. (2015)
		Inducing the expression of LTB4DH, and inhibiting the activity of MPO	ISO induced MI mice	8 mg/kg/day. Injectied for 3 days	*In vivo*	Suzuki et al. (2021)
	Anti-cerebral ischemia effect	Increasing passive avoidance memory and tail flick latency	BCCA induced I/R rats	50,100 or 200 mg/kg, orally for 5 days	*In vivo*	Farbood et al. (2013)
		Improving spatial memory in the Morris water maze	2VO induced VD rats	100 mg/kg, orally for 10 days	*In vivo*	Korani et al. (2014)
		Reducing inflammation related factors TNF- α Content and level of miR-124	Rats with PM + I/R	100 mg/kg, treated for 10 days	*In vivo*	Bavarsad et al. (2023)
		Activating microglia, reducing the levels of iNOS, COX-2, and MCP-1, and increasing the levels of Arg-1, CD206, and IL10	MCAO mice	150 mg/kg, injected for 3 days	*In vivo*	Qu et al. (2022)
		Inhibiting MPTP opening and mitochondrial membrane potential dissipation, and increasing ATP levels	Na₂S₂O₄-induced SH-SY5Y cells	0.1, 1, 10 μM, treated for 24 h	*In vitro*	Sun et al. (2014)
		Inhibiting the binding of CypD to ANT and enhancing ERK phosphorylation	H_2_O_2_ induced SH-SY5Y cells	0.1–10 μM,culture medium for 2 h	*In vitro*	Sun et al. (2017)
Negative effects of GA	Potential adverse effects	Losing weight and showing signs of anemia	F344 rats	357 mg/kg/day for males and 384 mg/kg/day for females, orally for 14 weeks	*In vitro*	Niho et al. (2001)
		Inhibiting PPAR-α level, activating the Ras/Raf/JAK/STAT pathway signaling pathway	CEM	2, 6, 10, and 14 μM for 1 day	*In vitro*	Hsieh et al. (2015b)
		Increasing fosab neuronal activity markers and cumulative motor responses, impairing GABA-glutamate balance	72 hpf zebrafish larvae	Exposured 30 min	*In vitro*	Annona et al. (2021)
		Reducing sperm output, sperm quality and testicular testosterone level	Rats	100 mg/kg/d, orally for 4 weeks	*In vitro*	Abarikwu et al. (2014)

## 6 Toxicity and adverse reactions

Acute toxicity tests indicated that albino mice with a single oral dose of 2,000 mg/kg of GA did not exhibit any toxicological conditions. Furthermore, subacute toxicity findings revealed that daily oral administration of 900 mg/kg of GA for 28 days did not significantly affect morphological and behavioral parameters, nor did it significantly alter hematological and histopathological parameters (Variya et al., 2019). In F344 rats, the administration of high doses of GA orally for a period of 14 weeks, at a dosage of 357 mg/kg/day for males and 384 mg/kg/day for females, resulted in the induction of significant toxic effects in both sexes. These effects included weight loss, signs of anemia (such as reduced red blood cell count), and pathological changes in the spleen, liver, and kidneys (Niho et al., 2001).

Furthermore, studies in both animals and cells had shown that high doses of GA potentially exhibited embryotoxic, neurotoxic, and reproductive toxicities. In the Chicken Embryo Model, concentrations of GA at or above 6 μM (1.02 mg/kg) found to induce significant embryotoxic effects, characterized by the dissolution of cerebral blood, degeneration of adipose tissue in neck muscles, and developmental disorders. These effects were linked to GA’s capacity to suppress levels of PPAR-α, elevate concentrations of NO, H2O2, and malondialdehyde, and activate the Ras/Raf/JAK/STAT signaling pathways. Moreover, supplementation with GSH, vitamin E, and N-acetylcysteine showed to alleviate these adverse reactions (Hsieh C. L. et al., 2015; Hsieh C.-L. et al., 2015). Furthermore, in zebrafish embryos (*Danio rerio*), GA (200 μg/mL) significantly inhibited embryo hatching and cardiac development (Harishkumar et al., 2019). Using zebrafish larvae that had hatched 72 h prior, brief exposure (30 min) to GA induced over-excitation of the central nervous system and behavioral changes in the larvae. This was characterized by increased activity of fosab neuronal markers and cumulative motor responses, and impaired GABA-glutamate balance, revealing GA’s potential neurotoxicity (Annona et al., 2021).

Furthermore, GA (25–100 μM) reduced the cell viability of mouse spermatogonia, mouse spermatocytes, and mouse Sertoli cells, suggesting that GA might have adverse effects on male reproductive health, which was associated with an increase in H2O2 levels (Park et al., 2008). The latest research showed that GA had reproductive toxicity by causing oxidative stress and inflammation. Studies have found that oral administration of GA (100 mg/kg/d) for four consecutive weeks reduced the quantity of sperm output, sperm quality, and testicular testosterone levels, while inhibiting the activity of steroidogenic enzymes (3β-HSD and 17β-HSD) and antioxidants (GSH, GPx, and SOD). Treatment of primary Sertoli cells with GA (25–100 μM) stimulated the expression of Tgf-β1 and CD-14, activated NF-κB and degraded IκBα, suggesting that it triggers inflammation through the NF-κB signaling pathway. Notably, curcumin can mitigate these adverse effects of GA (Abarikwu et al., 2014).

## 7 Discussion and conclusion

The extraction and synthesis of GA have laid a solid foundation for its widespread application. Research on GA extraction has developed various efficient methods (ultrasound-assisted, molecular blotting and supercritical CO_2_ extraction), significantly improving extraction efficiency by optimizing parameters such as solvent ratios, extraction temperature, and time (Baite et al., 2021; Khodaie and Ghoreishi, 2021; Pawar and Surana, 2010). In addition, the synthetic pathways of GA, including tannin degradation or synthesis via the shikimic acid pathway, have expanded the possibilities for its industrial-scale production. However, existing methods are costly and lack environmental sustainability, necessitating the development of more efficient, eco-friendly, and cost-effective technologies to meet industrial production demands.

Current studies indicated that GA has low bioavailability, primarily due to rapid metabolism and excretion in the gastrointestinal tract, with significant accumulation in organs such as the kidneys and liver. Repeated dosing and specific health conditions significantly alter its pharmacokinetics, highlighting the need for personalized dose adjustments (Ferruzzi et al., 2009; Yu X.-A. et al., 2018). However, researchers have synthesized GA derivatives and formulations to address these issues. The derivatives of GA, such as propyl gallate (E310), octyl gallate (E311) and dodecyl gallate (E312) are commonly used as preservatives in food and can be easily hydrolyzed to GA, helping to enhance the bioavailability of GA (Dhiman and Mukherjee, 2021). Studies showed that short-chain derivatives like E310 had higher hydrolysis and absorption efficiency, whereas long-chain derivatives such as E311 and E312 exhibited significantly lower absorption efficiency (Badhani et al., 2015; van der Heijden et al., 1986). Additionally, in Western societies, due to dietary habits, differences in gut flora, and their rapid metabolism and clearance, the actual bioavailability of GA and its derivatives was low (Randeni et al., 2024; Yang et al., 2020). This phenomenon suggested that further researches are needed to enhance GA’s absorption efficiency through optimized delivery systems, such as nanocarriers.

Studies have demonstrated that innovative formulations of GA, including nanoparticles, gels, colloids, nanoemulsions, and liposomes, significantly enhance its bioavailability, solubility, and transmembrane transport. For example, GA-loaded nanoparticles have been shown to prolong the half-life, increase plasma concentrations, and enhance overall bioavailability (Hassani et al., 2020; Patil and Killedar, 2021a; Y. Zhao et al., 2020b). Although advanced delivery systems, such as GA-loaded nanoparticles, hydrogels, and nanoemulsions, have significantly improved bioavailability and therapeutic outcomes, these technologies face challenges. Nanoparticle formulations involve complex preparation processes. Hydrogel and colloidal formulations are largely limited to topical use. Encapsulated compounds may interact with the encapsulating materials. And emulsions tend to be unstable. Therefore, future research should focus on optimizing these formulation technologies to improve their effectiveness and safety in clinical applications.

Atherosclerotic cardiovascular diseases (ASCVDs) are inflammatory conditions, manifested as aortic, coronary, and cerebrovascular diseases due to plaque instability or thrombosis. ASCVD is often associated with hypertension, diabetes, hyperlipidemia, and obesity, highlighting the importance of managing these risk factors for prevention (Nayor et al., 2021). Traditional treatments for cardiovascular sequelae typically include cholesterol-lowering drugs, antihypertensives, antiplatelets, thrombolytics, and anticoagulants. However, these treatments usually target specific conditions and may not address the broader spectrum of atherosclerotic complications, often leading to drug interactions and significant patient burden. Despite the variety of drugs available, there is still a lack of treatments that cover multiple atherosclerosis-related conditions (Bhatia et al., 2023; Ip et al., 2023; Kónyi et al., 2016).

GA, as a natural multi-target compound, is widely used in the food industry, aligning with the current trend towards developing safer, more comprehensive treatment strategies. Compared to traditional single-target drugs, GA possesses antioxidant, anti-inflammatory, blood lipid-regulating, blood sugar-reducing, and endothelial function-improving properties, allowing it to more comprehensively intervene in the pathological processes associated with acute cardiovascular diseases. This review explored the availability, pharmacokinetics, pharmacology, and safety of GA, which aimed to enhance its clinical applications. It also highlighted GA’s significant potential against ASCVD and related risk factors. Research indicated that GA exhibited preventive and therapeutic effects on ASCVD, including diabetes, obesity, hyperlipidemia, hypertension, atherosclerosis, cerebral ischemia, and myocardial infarction. Furthermore, the paper emphasized that GA’s pharmacological actions involved multiple key signaling pathways, such as PI3K/Akt, AMPK/Sirt1/PGC1α, ERK/CypD/NOX4/Poldip2, PKCα/p38MAPK, Akt/GSK3β, PI3K-Akt-ERK1/2, and AMPK-eNOS-FAS. Its effects were also associated with the regulation of ATGL, GLUT4, PPARα, PPARγ, NO, Ca^2+^, and MDA levels. Notably, PPARα and PPARγ, two nuclear receptors, play distinct roles in metabolic regulation. PPARγ is crucial for adipocyte differentiation and lipid storage, while PPARα is essential for lipid metabolism (Bougarne et al., 2018; Hernandez-Quiles et al., 2021). In HFD mice, GA suppressed the expression of PPARα and increased the expression of PPARγ, highlighting GA’s potential for metabolic disorders (Chao et al., 2020; Makihara et al., 2016). By influencing glucose metabolism and lipid homeostasis, GA emerges as a promising candidate for managing metabolic syndrome.

Furthermore, in a series of therapeutic studies targeting these metabolic diseases, literature reports indicated that the dosage of GA was usually high. For example, in these disease models, the dosage of GA ranged from 50 to 100 mg/kg per day, with treatment periods lasting from 4 to 24 weeks. This dosage was significantly higher than that used in studies for treating atherosclerosis, myocardial infarction, and cerebral ischemia, which ranged from 7 to 50 mg/kg over 3–10 days (Table 1). Although GA has shown potential in the prevention and treatment of ASCVD, there are still some limitations in the existing research. Future research needs to further explore whether GA can lower blood glucose through non-insulin-dependent pathways and its specific effects on different types of fat cells. Additionally, it is worth investigating whether GA involves other mechanisms in regulating blood pressure and the direct clinical evidence for GA treatment of arterial stiffness. In the context of atherosclerosis, the regulatory effects of GA on foam cells and macrophages require more detailed investigation, especially as the specific mechanisms of its anti-ASCVD actions have not been fully elucidated.

Acute toxicity studies showed that a single oral dose of GA up to 2,000 mg/kg in mice did not exhibit significant toxicity, suggesting that GA was sufficiently safe for the prevention and treatment of ASCVD (Variya et al., 2019). However, prolonged high doses (357–384 mg/kg over 14 weeks) led to cumulative toxicity (Niho et al., 2001). GA demonstrated notable embryotoxicity and neurotoxicity in animal embryo models such as chicken embryos and zebrafish (Annona et al., 2021; Hsieh C. L. et al., 2015). Current research, however, primarily focused on the toxic effects of short-term exposure. Additionally, the potential threat of high concentrations of GA (25–100 μM or 100 mg/kg/day) to male reproductive health, particularly its impact on male fertility, deserved special attention (Abarikwu et al., 2014). Further *in vivo* studies were needed to determine whether GA posed reproductive toxicity risks for females. In conclusion, GA has a relatively high safety profile, but potential risks associated with high doses or long-term use warrant attention. Future research should further evaluate its long-term safety and reproductive toxicity to ensure safe application.

In conclusion, GA, as a natural multi-target compound, demonstrates significant clinical value in the prevention and treatment of ASCVD, addressing the current demand for safer and more comprehensive therapeutic strategies. In particular, it is potentially beneficial for patients with metabolic disorders or those seeking complementary approaches to traditional drug therapy. Its antioxidant, anti-inflammatory, lipid-regulating, glucose-lowering, and endothelial function-improving properties enable a holistic intervention in ASCVD-related pathological processes, making it a promising adjunctive therapy to traditional treatments. Given the lack of a systematic review on the role and mechanisms of GA in ASCVD, this paper provides a comprehensive summary on the topic, offering valuable insights for future research and paving the way for its validation through mechanistic studies and clinical trials. With further evidence, GA is expected to emerge as a safe, affordable, and effective adjunctive therapy for ASCVD prevention and management.

## Glossary

**ASCVD**: Atherosclerotic cardiovascular disease
**AS**: atherosclerosis
**CHD**: coronary heart disease
**GA**: Gallic acid
**T2DM**: type 2 diabetes
**HOMA-IR**: Homeostasis Model Assessment of Insulin Resistance
**STZ**: streptozotocin
**SFRP4**: secreted frizzled-related protein 4
**IR**: insulin receptor
**IRS-1**: insulin receptor substrate 1
**PI3K**: Phosphoinositide 3-Kinase
**Akt**: Protein Kinase B
**NF-κB**: Nuclear Factor kappa-light-chain-enhancer of activated B cells
**IL-6**: Interleukin-6
**TNF-α**: Tumor Necrosis Factor-alpha
**GLUT2**: glucose transporter 2
**SGLT1**: sodium-dependent glucose transporter protein 1
**ACC**: acetyl-CoA carboxylase
**LDL**: low-density lipoprotein
**HFD**: high-fat diet
**TG**: triglyceride
**FAS**: Fatty Acid Synthase
**pAMPK**: phosphorylated AMP-activated protein kinase
**OA**: oleic acid
**NAFLD**: non-alcoholic fatty liver disease
**ATGL**: Adipose triglyceride lipase
**HSL**: hormone-sensitive lipase
**PPARγ**: Peroxisome Proliferator-Activated Receptor Gamma
**PPARα**: Peroxisome proliferator-activatedve receptor alpha
**BAT**: Brown adipose tissue
**Fas/FasL**: Fas Cell Surface Death Receptor/Fas Ligand
**Fabp4**: fatty acid-binding protein-4
**L-NAME**: Nω-Nitro-L-arginine methyl ester
**ERK**: Extracellular Signal-Regulated Kinase
**CypD**: Cyclophilin D
**HDAC1**: histone deacetylase 1
**HDAC2**: histone deacetylase 2
**HUVEC**: human umbilical vein endothelial cells
**HMEC-1**: human microvascular endothelial cells
**Nox**: NADPH Oxidase
**eNOS**: endothelial nitric oxide synthase
**LV**: left ventricle
**SHRs**: spontaneously hypertensive rats
**CaMKII**: calcium/calmodulin-dependent protein kinase II
**ISO**: isoproterenol
**I/R**: ischemia/reperfusion
ROS: Reactive Oxygen Species
**MDA**: malondialdehyde
**MPO**: myeloperoxidase
**SOD**: superoxide dismutase
**CAT**: catalase
**GPx**: glutathione peroxidase
**GST**: purinergic enzymes, detoxifying enzymes
**δ-ALA-D**: heme biosynthesizing enzymes
**LDH**: lactate dehydrogenase
**TBARS**: Thiobarbituric Acid Reactive Substances
**LTB4DH**: Leukotriene B4 12-Hydroxydehydrogenase
**PTEN**: Phosphatase and Tensin Homolog deleted on Chromosome 10
**RhoA**: Ras Homolog Family Member A
**Rac1**: Ras-related C3 Botulinum Toxin Substrate 1
**CDC42**: Cell Division Cycle 42
**CDK1**: cytokinin-dependent kinase 1
**TGF-β**: transforming growth factor-β
**LVDP**: left ventricular developed pressure
**±dp/dt**: rates of pressure development
**RPP**: rate-pressure product
**cTnT**: cardiac troponin T
**VD**: vascular dementia
**2VO**: permanent bilateral common carotid artery occlusion
**CHI**: cerebral hypoperfusion ischemia
**MCAO**: Middle Cerebral Artery Occlusion
**MPTP**: mitochondrial permeability transition pore
**MMPs**: matrix metalloproteinases
**BBB**: blood-brain barrier
**SDH**: shikimate dehydrogenase
**BCCA**: bilateral common carotid artery
**GSPE**: grape seed polyphenol extract
**Cmax**: maximum plasma concentration
**Tmax**: time to reach maximum concentration
**T1/2**: half-life
**AUC**: the area under the curve
**HPLC**: High-performance liquid chromatography
**LPHNs**: lipid-polymer hybrid nanoparticle system
**GANPs**: GA into nanoparticles
**4-Ω**: 4-methyl GA
**4-OMePCA**: 4-methyl protocatechuic acid
**MNPs**: Magnesium ferrite nanoparticles
**CS**: Hydrogels based on chitosan
**AMPS**: 2-acrylamido-2-methylpropane sulfonic acid
**GACNPs**: GA-based carbon nanoparticles
**FA**: ferulic acid
**HPβCD**: 2-hydroxypropyl-β-cyclodextrin
**SNEDDS**: self-nanoemulsifying drug delivery system
**SREBP-2**: sterol regulatory element-binding protein 2
**HMGCS**: β-hydroxy-β-methylglutaryl-CoA synthase
**TAG**: triglyceride
**PWV**: pulse wave velocity
**VSMCs**: vascular smooth muscle cells
**AGEs**: advanced glycation end products
**DNMT1**: DNA (Cytosine-5)-Methyltransferase 1
**PKCα**: Protein Kinase C alpha
**p38 MAPK**: p38 Mitogen-Activated Protein Kinase
**GSK3β**: Glycogen Synthase Kinase 3 beta
**CPK**: creatine phosphokinase
**CK-MB**: creatine kinase-MB
**4VO**: four-vessel occlusion.
